# Effectiveness of Lifestyle Nutrition and Physical Activity Interventions for Childhood Obesity and Associated Comorbidities among Children from Minority Ethnic Groups: A Systematic Review and Meta-Analysis

**DOI:** 10.3390/nu15112524

**Published:** 2023-05-29

**Authors:** George Obita, Ahmad Alkhatib

**Affiliations:** School of Health and Life Sciences, Teesside University, Tees Valley, Middlesbrough TS1 3BX, UK

**Keywords:** diet, behaviour, exercise, health programme, healthcare prevention, ethnicity, high-risk population

## Abstract

Lifestyle physical activity (PA) and nutrition are known to be effective interventions in preventing and managing obesity-related comorbidities among adult populations but less so among children and adolescents. We examined the effectiveness of lifestyle interventions in children from minority ethnic populations in Western high-income countries (HICs). Our systematic review included 53 studies, involving 26,045 children from minority ethnic populations who followed lifestyle intervention programmes lasting between 8 weeks and 5 years with the aim of preventing and/or managing childhood obesity and associated comorbidities, including adiposity and cardiometabolic risks. The studies were heterogenous in terms of lifestyle intervention components (nutrition, PA, behavioural counselling) and settings (community vs. schools and after-school settings). Our meta-analysis included 31 eligible studies and showed no significant effects of lifestyle interventions when they focused on body mass index (BMI) outcomes (pooled BMI mean change = −0.09 (95% CI = −0.19, 0.01); *p* = 0.09). This was irrespective of the intervention programme duration (<6 months vs. ≥6 months), type (PA vs. nutrition/combined intervention) and weight status (overweight or obese vs. normal weight) as all showed nonsignificant effects in the sensitivity analysis. Nonetheless, 19 of the 53 studies reported reductions in BMI, BMI z-score and body fat percentage. However, the majority of lifestyle interventions adopting a quasi-design with combined primary and secondary obesity measures (11 out of 15 studies) were effective in reducing the obesity comorbidities of cardiometabolic risks, including metabolic syndrome, insulin sensitivity and blood pressure, in overweight and obese children. Preventing childhood obesity in high-risk ethnic minority groups is best achieved using combined PA and nutrition intervention approaches, which jointly target preventing obesity and its comorbidities, especially the outcomes of diabetes, hypertension and cardiovascular disease. Therefore, public health stakeholders should integrate cultural and lifestyle factors and contextualise obesity prevention strategies among minority ethnic groups in Western HICs.

## 1. Introduction

Preventing the persistent rises in noncommunicable diseases (NCDs), such as cardiovascular disease, diabetes and cancer, is an immediate public health priority [1,2,3,4]. Obesity remains the main modifiable NCD risk factor, which has seen an alarming increase globally and is now associated with reduced life expectancy [5]. Prevalence estimates have shown concurrent increases in obesity, physical inactivity and poor dietary quality and patterns across all age groups [6,7,8,9]. Recent post-COVID-19 reports have estimated that over 380 million children are currently overweight or obese worldwide [10,11]. Alarmingly, this age group is also at risk of imminent rises in childhood obesity-related comorbidities, including hypertension, insulin insensitivity, fatty liver, type-2 diabetes (T2D) and cardiovascular disease (CVD) [12].

There is established evidence in adult populations of the benefits of lifestyle interventions in preventing obesity-related cardiometabolic diseases, such as diabetes [13]. Although the effectiveness of lifestyle interventions in children is not well established [14,15], recent reviews and meta-analyses have concluded that overall, lifestyle interventions combining physical activity and nutritional modifications represent the most promising means for preventing childhood obesity [16,17]. However, these reviews have also highlighted that high-risk population groups, including those from ethnic minorities, who have increased risks of obesity and associated diseases are not targeted effectively with such interventions, especially at the community level [17]. For example, children from ethnic minority groups with low socioeconomic status have higher risks of obesity and healthcare disparity [18,19]. Consequently, the likelihood of obese children from high-risk minority groups developing comorbidities, such as fatty liver, hypertension, T2D and CVD, is increased. It has recently been shown that children from ethnic minority groups who have higher rates of obesity are more likely to be exposed to both NCDs and worse COVID-19 outcomes [10,20]. We previously reported a disparity in the prevalence of childhood obesity and related comorbidities, especially due to ethnicity and social inequality determinants [21]. Yet the available knowledgebase of effective intervention approaches to guide the development of childhood obesity and NCD prevention interventions targeted towards those at greatest risk remains limited [22,23,24]. 

The effective lifestyle interventions of improving dietary quality, physical activity (PA) levels and sedentary behaviour are known to ameliorate obesity and associated NCDs in children [25,26,27]. However, most studies have been conducted in predominantly white populations [27,28]. It has often been assumed that lifestyle interventions found to be effective in the general population are likely to be effective among ethnic minority populations, if appropriately adapted [28]. However, the discrepant effectiveness of behavioural interventions among different population groups has been reported [29,30]. Furthermore, there is currently limited evidence to prove or disprove the effectiveness of adapted behavioural interventions in preventing childhood obesity among minority ethnic groups [31]. Therefore, it has been suggested that targeted interventions are likely to be more effective than universal approaches in this circumstance because of the unique barriers and inequities faced by minority ethnic groups [32]. Moreover, risk stratification and targeted interventions have been shown to play a role when high-risk groups face unique barriers [33]. 

Therefore, it is important to identify lifestyle intervention approaches that are likely to be effective among children from minority ethnic groups in Western high-income countries (HICs), given the reported disparity in prevalence between HICs and low- and middle-income countries (LMICs) [21]. There are currently no reviews or analyses on whether or how lifestyle interventions are effective in preventing childhood obesity-related comorbidities among high-risk minority ethnic groups. Therefore, this systematic review and meta-analysis aimed to assess the effectiveness of lifestyle interventions among minority ethnic groups living in Western HICs and describe the salient features of effective interventions for preventing childhood obesity.

For the purpose of this review, the term minority ethnic groups is used to describe people of non-White descent living in Western HICs, in accordance with the common terminology applied in the UK [34]. 

## 2. Materials and Methods

The protocol for this review was registered with the International Prospective Register of Systematic Reviews (PROSPERO: CRD42022369557) and followed the Preferred Reporting Items for Systematic Reviews and Meta-Analyses (PRISMA) [35]. 

### 2.1. Search Strategy

We used the Patient/population Intervention Comparator and Outcome (PICO) framework to develop the search strategy [36] and conducted systematic searches of different electronic databases using a combination of free text and medical subheadings (MeSH) to locate published studies. We searched the MEDLINE, EMBASE, CINAHL, PsycInfo, SCOPUS, SPORTDiscus and Cochrane Controlled Trials Register databases and conducted manual searches using the lists of references from relevant studies. Furthermore, we searched the registers of controlled trials in progress, conference proceedings and the general Internet using Google Scholar. Initially a specific search strategy for MEDLINE (see Supplementary Materials Table S1) was developed with the help of a Teesside University librarian. This was then adapted to the other databases.

The following search terms were used: [(children or adolescents or paediatric or students or school pupils or youth or boys or girls or school age or juvenile or preteens or teens) AND (BME or BAME or Black and minority ethnic group or Black African or Indian or Pakistani or Bangladeshi or Chinese or mixed race or Hispanic)] AND [(physical activity or exercise or sport or cycling or walking or physical education or aerobics or fitness class/regime/programme or dance therapy or intervention for or sedentary lifestyle) OR (diet therapy/diets/dieting or fasting or healthy eating or fruit or vegetable or formula) OR (behaviour therapy or social support or psychotherapy, group or family therapy or counselling or social support or peer support or health education/health promotion or media intervention or community intervention school programme or health policy on food or nutrition)] AND [(obesity or body weight or adiposity or body mass index or waist circumference or neck circumference) OR (type 2 diabetes mellitus or hypertension or high blood pressure or cardiovascular disease or CVD or metabolic syndrome or non-alcoholic fatty liver disease or NAFLD or depression or psychological problem or anxiety or self-esteem or sleep apnoea or asthma or respiratory problem or dyslipidaemia or musculoskeletal problem)]. The searches were filtered for randomised controlled trials (RCTs) and quasi-RCT study designs. 

### 2.2. Inclusion and Exclusion Criteria for Studies

As the review examined lifestyle interventions for the prevention of overweight/obesity and related NCDs among children from minority ethnic groups living in Western HICs, studies were included if (i) they were quasi-randomised studies or RCTs that compared lifestyle interventions with no intervention or other interventions with the primary aim of preventing or managing obesity and associated NCDs as these are the most appropriate study designs for determining effectiveness [37], (ii) they included 0 to 18 year old children, with minority ethnic groups constituting the majority (at least 60%) of the study participants, (iii) they used lifestyle interventions, such as physical activities, diet and reductions in sedentary activity, to prevent obesity and associated NCDs, (iv) they included the outcomes of interest of adiposity measures and metabolic risk factors of NCDs, (v) they were set in Western HICs and (vi) there were no restrictions on timing or language, provided the studies could be translated into English using Google Translate.

Studies were excluded if (i) they were of other designs (such as cohort studies, case-control studies, cross-sectional studies, case series or case reports), (ii) the participants were adults or from the general population without minority ethnic children being the main target group, (iii) they used lifestyle interventions for other outcomes and not obesity or NCD prevention or management or (iv) they were conducted in regions other than Western HICs (see Supplementary Materials).

### 2.3. Study Selection, Quality Assessment and Data Extraction 

EndNote reference management software version 9, 2019, was used to upload and remove duplicates and share articles identified from the searches between reviewers. 

#### 2.3.1. Selection Process

**Figure 1:**
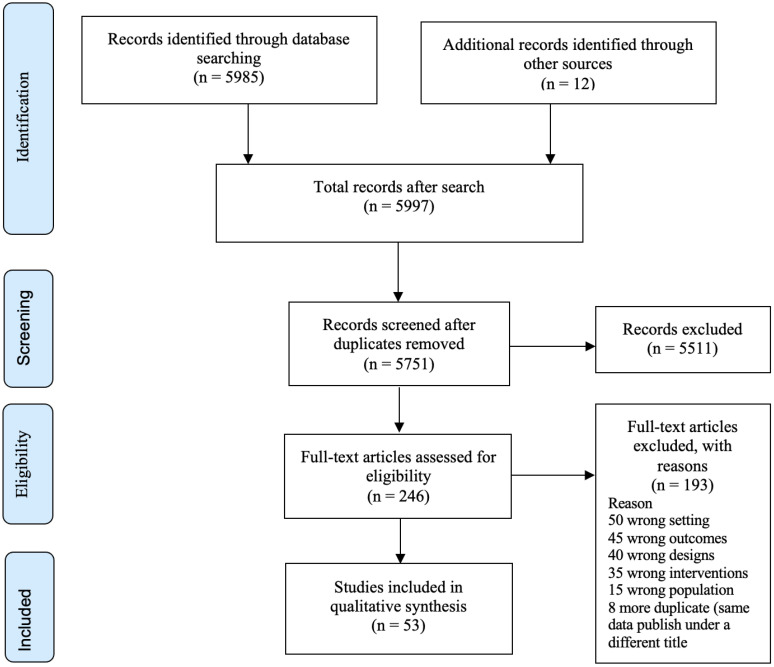
A flowchart of the study selection process, based on the PRISMA guidelines.

Two reviewers independently screened all of the title/abstracts of the identified studies against the inclusion and exclusion criteria (see Supplementary Materials Table S2). One reviewer then retrieved the full papers of the selected articles and two reviewers reviewed them in detail. The reasons for excluding any full text studies were documented. The search results were reported according to the Preferred Reporting Items for Systematic Reviews and Meta-analyses (PRISMA) flow diagram (Figure 1) [38].

#### 2.3.2. Risk of Bias in Individual Studies

In this review, the risk of bias for all selected studies was assessed using the Cochrane risk of bias tool [39]. Unlike most tools used for assessing the quality of studies in the context of systematic reviews, the Cochrane tool is not a scale or a checklist [40]. It is a domain-based evaluation that allows for the critical assessment of different domains in RCTs [39]. Given that it is impossible to quantify bias in a given study, this tool allows for qualitative and quantitative judgments and, therefore, is more realistic than checklist- or scale-based tools [41].

Two reviewers independently determined the risk of bias in the individual studies using the Cochrane risk of bias tool (see Supplementary Materials Table S3). Six domains of the study designs and reporting were assessed: random sequence generation for randomisation, allocation concealment, the blinding of participants and personnel, the blinding of outcome assessment and selective reporting. With this tool, studies were classified as having a high, unclear (where the domains were not clearly described) or low risk of bias [39].

#### 2.3.3. Data Extraction Process (Selection and Coding)

One reviewer extracted data from the studies included in the review using a data extraction checklist and a second reviewer examined the extracted data (see Supplementary Materials Table S4). Any dispute was resolved through discussion. Data regarding study population, methodology, intervention/comparator details, outcome and main results were extracted and tabulated for analysis. 

### 2.4. Data Synthesis

We carried out a narrative synthesis of the results of both RCTs and quasi-experimental studies to compile data and identify common patterns. We compared the results of studies that used direct or indirect lifestyle interventions, such as counselling, to prevent or manage obesity and associated NCDs among children. We also compared the outcome, type of intervention, intervention duration and setting of intervention. Ultimately, the data synthesis teased out intervention approaches that were effective among children from minority ethnic groups and those that were not effective. Both tables and text were used to summarise these findings. 

### 2.5. Statistical Analysis 

We conducted the meta-analysis using Review Manager (RevMan) 5.4 [42], where appropriate. For example, when complete pre-post measures, such mean BMI/BMI z-score, sample size, standard deviation or standard error, were available for the intervention and control groups, the RCT was included in the meta-analysis. The first step in the meta-analysis was to assess the mean differences (MDs) in the outcomes for both the intervention and control groups by comparing changes in the means as the difference between the post-intervention and baseline measures. To calculate the MDs, the available adjusted or unadjusted means, as reported in the included studies, were used. The corresponding changes in standard deviation (SD) were not directly reported in most studies; therefore, these were estimated using the formula suggested by the Cochrane handbook for the systematic review of interventions [43]. Where standard error (SE) was reported, this was converted into SD using the following formula: SD ( = SE * √n (where SD = standard deviation, SE = standard error, n = sample size) [43]. The second step involved estimating the pooled effect for outcomes, where at least two RCTs reported the same outcome variables. The gain in the intervention group against the change in the comparator group was reported as the pooled effect, which was estimated with 95% CIs. The study weights were equal to the inverse of the variance of the estimated effect of each study, as suggested by DerSimonian and Laird [44]. The overall effect was interpreted as statistically significant if the 95% CI did not include the null value of 0 (no difference) in its range. Sensitivity analyses were performed to assess whether the correlations of 0.5 or 0.8 affected the interpretation of the pooled effect. Heterogeneity, i.e., variations in the intervention effects observed in the included studies, was quantified using the *I*^2^ statistic. Results are to be interpreted with caution where there was significant heterogeneity (*I*^2^ > 50%). 

## 3. Results

### 3.1. Study Selection

A total of 5751 unique articles were identified through the search process after removing duplicates. Following the title and abstract screening, we examined 246 full texts for eligibility. Of the full texts examined, 53 articles met our inclusion criteria (Figure 1). 

### 3.2. Characteristics of Included Studies 

We list the details of each of the included studies in Table 1. All but one of the included studies were conducted in the USA. The study that was not conducted in the USA was carried out in the United Kingdom (UK) [45]. Of the 53 included studies, 44 were RCTs and 9 were quasi-experimental pre-post designs. There were 19 studies conducted in school settings, 12 in community and home settings, 11 in more than one setting (such as school-based activities with homework or parental involvement and clinic and home settings), 9 in healthcare settings (such hospitals and primary healthcare and paediatric clinics) and 3 were web/online-based. 

A total of about 26045 participants took part in the studies, with an average sample size per study of 477 and a range of 17 to 4044. About 62% of the included studies had participants who were predominantly Hispanic Americans, 13% of the studies had predominantly African American participants, 9% of the studies had participants of mixed ethnic minority groups and four studies had Asian Americans as their main participants. The age of the participants ranged from 0 (new-born infants to mothers who participated in interventions) [46] to 18-year-old adolescents. Female participants comprised 44% of the total sample and 45% of studies targeted participants who were either overweight or obese (BMI ≥ 85th United States CDC BMI percentile for age and sex).

Most intervention programmes (77%) were a combination of nutrition, physical activity and behavioural interventions. PA alone comprised 13% of interventions, while nutrition alone and general behavioural interventions comprised 5% each. The majority (64%) of the interventions were implemented for 6 or more months, whereas 36% were implemented for less than 6 months. The duration of implementation varied from 8 weeks to 5 years. There were three RCTs [47,48,49] that had more than one intervention group. There were 18 of the 53 included studies that reported theoretical frameworks or models for the interventions. These included social cognitive theory (n = 8), a socioecological approach (n = 3), a transtheoretical model (n = 3), the Chronic Care Model (CCM) (n = 1), behaviour change theory (n = 1), social contextual change (n = 1), self-efficiency (n = 1) and a health belief model (n = 1).

The type of control or comparison group varied across the studies (Table 1). Overall, 52% of the RCTs compared interventions using ‘standard intervention’/‘usual care’, whereas 48% compared interventions with relatively more active comparisons, such as school-readiness programmes, self-esteem programmes, health and safety programmes, general health programmes and self-help programmes. 

Most of the included studies (n = 49) targeted obesity prevention (primary and secondary) as the main outcome. There were 18 studies that targeted cardiometabolic NCD risk factors, such as insulin resistance, hyperglycaemia, hyperlipidaemia and high blood pressure, either as primary or secondary outcomes. The most used measures of adiposity were zBMI and BMI. Only 12 studies only used BMI as the measure of adiposity, whereas 9 studies only used zBMI as the measure of adiposity and 6 studies used a combination of BMI and zBMI as the measures of adiposity. Seven studies used BMI in combination with other measures of adiposity, such as percentage body fat, waist circumference (WC) and waist–hip ratio. Twelve studies combined measures of adiposity and cardiometabolic outcomes, whereas four studies only used measures of cardiometabolic risk factors as the primary outcomes.

**Table 1:** The characteristics of intervention studies for obesity and associated comorbidity prevention in children from minority ethnic populations.

Study	Study Design, Setting and Sample	Study Participants (Sample, Ethnicity and Age)	Intervention (Type, Duration, Frequency and Theory Base)	Comparator Control	Main Results	Comments on Key Strengths/Limitations
Yli-Piipari et al., 2018, USA [50].	Quasi-experimental one-arm pre- and post-test design, conducted in a primary care setting.	22 high-risk overweight and obese Hispanic children (BMI ≥85th United States CDC BMI percentile for age and sex); Mean age 11.7 years; 27% female.	12 weeks PA and nutrition behaviour programme: 60 min twice per week (for a total of 24 h) of moderate- to vigorous-intensity boxing exercise, 12 h of nutrition education for guardians and a 30-min paediatrician appointment.	None	BMI (kg/m^2^) change: t(15) = −2; BMI% change: t(15) = −2.53, *p* = 0.023, d = 0.20, *p* = 0.044, d = 0.5; BM z-score change: t(15) = −3.64, *p* = 0.002, d = 0.19; WC change: t(17) = −2.57, *p* = 0.020; Fasting glucose change: t(15) = −6.43, *p* < 0.001, d = 1.67.	In Hispanic children with severe obesity, the multicomponent supervised exercise and nutrition intensive programme was effective in the short term, reducing obesity and metabolic risk (fasting glucose). However, long-term adherence to this programme is unknown.
Yin et al., 2012, USA [49].	Quasi-experimental pre- and post-test design with two groups, conducted in community Head Start centres and home settings.	384 predominantly Hispanic children; 52% female; attending community Head Start centres; aged 3 to 5 (mean = 4.1) years.	18-week PA and nutrition intervention. Centre-based intervention: (i) PA: 60 min of structured (15–20 min) and free play (30–45 min) per day; (ii) Nutrition promotion. Home-based intervention: (i) Peer-led parent obesity education; (ii) Healthy snacks for their children (<150 calories).	The control group received the intervention materials and implementation training upon completing the study.	Adjusted difference in BMI z-score for age and gender between centre-based + home-based intervention and comparator = –0.09, *p* < 0.09; Adjusted difference in BMI z-score for age and gender between comparison and centre-based intervention = −0.04 (not significant).	This large study with intervention in both centre and home settings targeting both PA and nutrition showed improvements in BMI z-scores, though not statistically significant. The participants were children not described as overweight or obese; therefore, nonsignificant reductions in zBMI were to be expected.
Yin et al., 2005, USA [51].	Quasi-experimental pre- and post-test design with two groups, conducted in an elementary school setting.	601 predominantly Black (61%) elementary school children; Mean age 8.7 years; 52% female.	24-week (8-month) after-school programme: (i) 40 min academic enrichment; (ii) Healthy snack; (iii) 80 min of PA.	265 children served as the control, who only received health screening (no after-school activities).	BMI (kg/m^2^) change: −0.16 (−0.40, 0.07), *p* = 0.18; % Body fat (BF): 0.76 (1.42, 0.09), *p* = 0.027; Fat mass (FM) (kg): −0.29 (−0.70, 0.13), *p* = 0.17; Free fat mass (FFM) (kg): 0.18 (−0.04, 0.40), *p* = 0.12; WC (cm): −0.4 (−1.1, 0.4), *p* = 0.32; SBP (mmHg): −1.8 (−4.2, 0.6), *p* = 0.15; DBP (mmHg): −1.1 (−3.6, 1.5), *p* = 0.41; TC (mg/dl): −0.2 (−6.2, 5.7), *p* = 0.94; HDL (mg/dl): 0.7 (−2.1, 3.5), *p* = 0.64.	After-school intervention programme had some effects on BMI, body fat and lipid profile in Black communities, but not statically significant. The interventions were difficult to adhere to in home and community settings because they lacked parental involvement.
Wylie-Rosett et al., 2018, USA [52].	RCT, conducted in a safety-net paediatric primary care setting in the Bronx, New York.	360 predominantly Hispanic (73%) children with BMI ≥85th United States CDC BMI percentile for age and sex; aged 7 to 12 (mean = 9.3) years; 33% female.	A 12-month 8-weekly programme: (i) Standard care; (ii) Enhanced programme (skill building core: food preparation or other skill activity for parents/guardians and children, PA session for the children and discussion session for parents/guardians regarding their role in weight management + post-core programme support)	Standard care participants had quarterly visits to see a paediatrician for weight management.	BMI Z-score change: The mean BMI Z-score decreased in both programmes (0.12 kg within the standard care group (*p* < 0.01) and 0.15 kg within both standard care + enhanced care groups (*p* < 0.01). No significant differences between the two programmes. Older children had a greater decline in BMI Z-score than younger children (beta = −0.04 units per additional year of age; *p* < 0.01). Girls exhibited a greater decline in BMI Z-score than boys (β = 0.09; *p* = 0.03). TC (mmol/L) change: −0.1, *p* = 0.05; HDL (mmol/L) change: 0.01, *p* = 0.67; LDL (mmol/L): −0.07, *p* = 0.04; Triglyceride (mmol/L): −0.06, *p* = 0.08.	In high-risk overweight/obese children, the enhanced care programme was not more effective than the standard care programme, although the clinical care in both groups reduced weight and improved lipid profiles.
Wong et al., 2016, USA [53].	A non-randomised trial, conducted in community centres located in low-income neighbourhoods within the city.	877 Hispanic and African American children; aged 9 to 12 years; 47% female.	9-month programme: (i) 90 min of structured PA twice a week for 6 weeks in the fall, early spring and at the end of the school year; (ii) 30 min of nutrition or healthy habits lessons twice a week during each of the three 6-week sessions.	Regular after-school childcare enrichment programmes at community centres offered by the site staff, such as homework time, arts and crafts activities and supervised free play.	There were no significant intervention effects on BMI (*p* = 0.94), BMI z-score (*p* = 0.88) or BMI percentile (*p* = 0.23).	Structured 90 min PA plus nutrition education was not more effective than supervised free play in reducing weight but helped to enhance regular exercise.
Wilson et al., 2022, USA [54].	RCT; Online setting.	241 African American child/care giver dyads; Children aged 11 to 16 years, with BMI ≥85th United States CDC BMI percentile for age and sex.	24-week (6-month) programme: (i) 8-week tailored online education on parenting, nutrition, PA and decreasing screen time. This was followed by 3 online booster sessions, 1 every 2 months.	Control online programme	There were no significant intervention effects on BMI but there was a significant effect of the group intervention on light physical activity among the parents at 16 weeks (*B* = 33.017, *SE* = 13.115, *p* = 0.012) and a similar trend for adolescents.	The online programme was not effective for BMI but had a useful impact on physical activity. Actual data were not shown on BMI mean differences between intervention and comparators for both children and parents.
Williford et al., 1996, USA [55].	Quasi-experimental with pre- and post-test analyses, conducted in a school setting.	17 African American male children in 7th grade from a physical education class; Aged 11 to 13 (mean = 12.8) years.	15-week programme: 5 days/week for 45 min session of PE class + conditioning programme (aerobic training on 3 days and weight training on 2 days).	PE class as usual	Sum of 7 skin fold thickness (mm): 99.01 ± 67.8 to 97.7 ± 67.4, *p* = 0.09; TC (mmol/L): 4.03 ± 0.81 to 4.03 ± 0.77, *p* = 0.98; HDL (mmol/L) change intervention group: 1 ± 0.18 to 1.28 ± 0.17, *p* < 0.05; LDL (mmol/L) change intervention group: 2.73 ± 0.74 to 2.41 ± 0.81, *p* < 0.05	Small improvement in HDL and LDL from the PA intervention. However, the sample was small and it was not clear how effective this PA alone intervention was on overweight/obesity.
Williamson et al., 2006, USA [56].	RCT; Internet-based interactive behaviour therapy.	57 African American girls; Aged 11 to 15 (mean = 13.2) years, with BMI >85th percentile for age and gender based on 1999 National Health and Nutrition Examination Study normative data and with a biological parent with BMI > 30.	A 96-week (24-month) Internet programme: (i) An interactive behavioural Internet programme; (ii) Face-to-face sessions and e-mail correspondence from a counsellor.	An Internet health education programme (a passive (non-interactive) programme that provided useful health education for parents and adolescents through electronic links to other health-related websites.)	BMI, F (3,54) = 3.13, *p* < 0.04; BF % change: 0.08 ± 0.71 vs. 0.84 ± 0.72 BF, *p* < 0.05.	The Internet-based intervention was effective in reducing weight in overweight/obese girls. However, the girls appeared to be a highly motivated group as they were willing to purchase their own computers for at least $300.00.
Van der Heijden et al. 2010, USA [57].	Quasi-experimental with pre- and post-test analyses; recruitment was conducted in a community setting, while checks for good health were carried out in hospital.	29 Hispanic adolescents; Median age 15 years, obese and lean (obese participants had BMI > 95th and lean participants had BMI <85th percentile for age, according to CDC growth chart; 48% females.	A 12-week PA programme supervised by an experienced exercise physiologist: (i) 30 min aerobic exercise session at ≥70% of peak oxygen consumption (VO_2peak_) twice a week at a hospital physical therapy unit.	None	In obese participants, intramyocellular fat remained unchanged, whereas hepatic fat content decreased from 8.9 ± 3.2 to 5.6 ± 1.8% (*p* < 0.05) and visceral fat content from 54.7 ± 6.0 to 49.6 ± 5.5 cm^2^ (*p* < 0.05). No significant changes were observed in lean participants. Insulin resistance: Decreased fasting insulin (21.8 ± 2.7 to 18.2 ± 2.4 μ/mL, *p* < 0.01) and homeostasis model assessment of insulin resistance (HOMAIR) (4.9 ± 0.7 to 4.1 ± 0.6, *p* < 0.01). No significant changes were observed in lean participants.	Aerobic exercise in a controlled environment reduced hepatic fat, visceral fat and insulin resistance in obese participants. The sample was small and involved selected individuals with severe adiposity; therefore, the results may not be generalisable.
Tomayko et al., 2018, USA [58].	A modified crossover design, conducted in 4 tribal reservations and 1 urban clinic setting.	450 American Indian adult/child dyads; Children were aged 2 to 5 (mean = 3.3) years; 50% female.	A 52-week (12-month) programme: Monthly mailed healthy lifestyle lessons, items and children’s books addressing six targets: increased fruit and vegetable consumption, decreased sugar consumption, increased PA, decreased screen time, improved sleep habits and decreased stress (adults only).	Active control-crossover.	BMI z-score at 1 year: Intervention = 0.80 ± 1.10; Comparator = 0.76 ± 1.04, *p* = 0.513.	The unsupervised mailed education materials were not effective in reducing BMI z-scores. The extent to which the materials were used was unknown.
Taveras et al., 2017, USA [59].	RCT, conducted in 6 paediatric practices in an urban setting.	721 predominantly (65%) non-White children; Children aged 2 to 12 (mean = 8) years, with BMI ≥85th percentile for age, according to CDC growth chart; 51% females.	A 12-month programme: Enhanced primary care plus contextually tailored individual health coaching lasting 15–20 min via telephone, videoconference (Vidyo) or in-person visits.	Enhanced primary care Two monthly educational materials focusing on healthy lifestyle behavioural changes.	BMI z-score: In the enhanced primary care group, there was an adjusted mean (SD) BMI z-score improvement of −0.06 BMI z-score units (95% CI = −0.10 to −0.02) from baseline to 1 year. In the enhanced primary care plus coaching group, there was an improvement of −0.09 BMI z-score units (95% CI = −0.13 to −0.05). However, there was no significant difference between the 2 intervention arms (difference = −0.02; 95% CI = −0.08 to 0.03; *p* = 0.39).	Advanced clinical care improved BMI z-scores in high-risk children (overweight/obese), but additional individual coaching did not add any effect.
Story et al., 2012, USA [60].	RCT, conducted in schools in reservations.	454 American Indian children attending Kindergarten and first grade; Mean age 5.8 years; 49% female.	A 45-week programme: (i) PA: school-based PA of at least 60 min daily; (ii) Nutrition: healthy eating at school; (iii) Family-focused intervention: improving nutrition and PA and reducing sedentary lifestyle; (iv) Parents received telephone motivational encouragement.	Usual school activities and no change in family environment.	Mean BMI (kg/m^2^) net difference (I vs. C): 0.34, *p* = 0.057; zBMI net difference: 0.01, *p* = 0.904. %BF net difference: 0.9, *p* = 0.122; Prevalence of overweight (BMI ≥85th percentile and <95th): net difference: 10.14, *p* = 0.019; Prevalence of obesity (BMI ≥95th percentile): 2.11, *p* = 0.503.	Interestingly, this multicomponent programme reduced the prevalence of overweight children, although participants were young children and not described as overweight.
Stolley et al., 2003, USA [61].	RCT, conducted in public school settings.	618 African American preschool children; Aged 3 to 5 (mean = 4.3) years; 53% female.	A 14-week programme: (i) Education: two lessons each week on healthy eating and exercise; (ii) Two 20 min session of PA each week; (iii) Parents received a weekly newsletter.	Usual preschool activities.	Adjusted BMI (kg/m^2^) difference: −0.08, *p* = 0.28; Adjusted BMI z-scores: −0.05, *p* = 0.23.	This predominantly nutrition and PA education intervention reduced BMI z-scores but was not more effective than usual school activities.
Soltero et al., 2018, USA [62].	RCT, recruitment conducted through schools, community centres and healthcare organisations but intervention was administered at YMCA centres.	160 Hispanic children; Aged 14 to 16 years, with BMI ≥95th percentile for age and sex, according to CDC growth chart, or a BMI ≥ 30 kg/m^2^; 46% females.	A 52-week (12 months) programme: (i). Nutrition and health education one days/week, 60 min. (ii). PA: exercise curriculum was delivered by fitness instructors three days/week for 60 min (iii). Behaviour changes strategies.	Handout with general information on healthy lifestyle behaviours.	Changes in insulin sensitivity (using insulin and glucose sensitivity during OGTT): Intervention: 0.8 ± 0.1 to 2.2 ± 0.1, *p* < 0.01; Comparator: 1.7 ± 0.2 to 1.7 ± 0.1, *p* > 0.05. Between-group difference (delta difference) Δ = 0.37, *p* < 0.05, at 12 weeks; Δ = 0.21, *p* > 0.05, at 12 months (no difference). Within-group changes in intervention group at 12 months: BMI (kg/m^2^) = 1.16, *p* < 0.001; BMI% = −0.1, *p* = 0.95; %BF = −0.63, *p* = 0.65; WC (cm) = 1.68, *p* = 0.29. At 12 months, between-group differences in BMI and percentage body fat remained significant (all *p* < 0.01); however, changes in WC was not (*p* = 0.078).	This seemed to be effective in increasing insulin sensitivity in the short term but there was no difference in the long term in this high-risk group with obesity. The intervention was shown to be effective in reducing adiposity parameters, which were sustained at 12 months. This long duration nutrition education and PA intervention improved insulin resistance, but only in the short term.
Slusser et al., 2012, USA [63].	RCT, conducted in a family clinic and wellness centre and community sites, serving a low-income predominantly Hispanic community.	161 Hispanic children; Aged 2 to 4 years, living in the home.	A 17-week programme: 9 sessions lasting 90 min of parent training, based on social learning theory.	Care as usual and a standard nutritional informational pamphlet.	BMI percentile changes: Intervention = −3.85; Comparator = 1.33. BMI z-score difference between intervention group and control: −2.4, *p* = 0.04. Children in the intervention group decreased their BMI z-scores significantly; on average, by 0.20 (se = 0.08) compared to children in the control group, who increased z-scores on average by 0.04 (se = 0.09) at 1 year (*p* < 0.05).	Only 9 sessions of parent training over 17 weeks were effective in reducing overweight/obesity in preschool children from low-income families. It was not clear whether this would be the same in a larger population or over a longer period.
Shaibi et al., 2006, USA [64].	RCT; Participants were recruited through medical clinics, advertisements and local schools, while the intervention was conducted at girls’ and boys’ clubs.	22 Hispanic male adolescents; Mean age = 15.3 years, with BMI ≥85th percentile for age, according to CDC growth chart.	A 17-week programme: (i) resistance training twice per week.	Non-exercising control group.	Changes in insulin sensitivity (×10^−4^ min^−1^ mL^−1^, using insulin and glucose sensitivity during OGTT): I = 0.9 ± 0.1, *p* < 0.05; C = 0.1 ± 0.3. The intervention group significantly increased insulin sensitivity compared to the comparator group (*p* < 0.05).	Resistance training alone significantly reduced the metabolic risk factor of insulin sensitivity within 3 months in overweight/obese children. However, its effect on adiposity was not reported.
Robinson et al., 2021, USA [65].	RCT, recruitment was conducted through medical clinics, advertisements and schools, while the administration of the intervention was carried out at the Los Angeles Boys’ and Girls’ Club.	241 primarily Hispanic children; Aged 7 to 11 years, with BMI ≥85th percentile for age and sex, according to CDC growth chart; 56% females.	A 3-year community-based, multilevel, multi-setting, multicomponent programme: (i) Home environment changes and behavioural counselling; (ii) Community after-school team sports; (iii) Reports to primary health-care providers.	General health education.	Mean adjusted difference in BMI trajectory over 3 years between multicomponent and HE = −0.25 (95% CI = −0.90, 0.40) kg/m^2^, Cohen’s *d* = −0.10, *p* = 0.45.	The multicomponent and multilevel intervention did not reduce BMI gain in low-socioeconomic overweight Hispanic children, despite the long duration of intervention. However, there was a drop in participation over time.
Rieder et al., 2013, USA [66].	Quasi-experimental with pre- and post-test analyses, conducted in a community setting.	349 majority minority ethnic group (52% Black and 44% Hispanic); Mean age = 15 years; 54% females.	A 9-month programme: (i) Teaching healthy lifestyle principles; (ii) 60 min per week of moderate PA; (iii) Monthly family healthy behaviour education.	No comparator intervention.	Decreases in BMI (kg/m^2^) (−0.07 per month; *p* < 0.001); Percent overweight (−0.002%/month; *p* < 0.001); BMI z-score (−0.003/month; *p* < 0.01); Decrease in BMI percentile (−0.006 percentile/month; *p* = 0.06).	This 9-month education and PA showed a small effect in reducing overweight/obesity in adolescents. However, their pre-intervention weights were unknown.
Resnicow et al., 2005, USA [67].	RCT, conducted in churches in a rural setting.	147 African American female children; Aged 12 to 16 years, with BMI >90th percentile for age and sex, according to CDC growth chart.	A 26-week (6-month) multicomponent programme tailored to the population: High-intensity PA (24 to 26 sessions). (i) At least 30 min of moderate to vigorous PA; (ii) Preparation and/or consumption of low-fat, portion-controlled meals or snacks; (iii) Parental involvement.	Moderate-intensity intervention. Six sessions of education, with topics including fat facts, barriers to physical activity, fad diets, neophobia (i.e., the fear of new foods) and the benefits of PA.	0.5 BMI units of difference. This difference was not statistically significant (*p* = 0.20).	There was no difference between high-intensity and moderate-intensity PA over 6 months in the group of obese African American adolescent girls. However, both groups showed some improvements in adiposity.
Prado et al., 2020, USA [68].	RCT, conducted in a community setting.	22 Hispanic children; Mean age = 13.1 years (in 7th/8th grade), with BMI >85th percentile for age and sex, according to CDC growth chart; 88% females.	A 12-week programme of 2.5-h sessions: 1.5 h of lifestyle education, involving families and children, and 1 h of PA for the children. PA was supervised by a coach in local park.	Prevention as usual. Participants were referred to their local health department’s health initiative Internet page and the usual programmes they offer to reflect the typical services that overweight and obese adolescents may receive in their own community.	BMI (kg/m^2^) difference between baseline and 2 years: −0.3 (95% CI = −0.7 to 0.1), *p* = 0.15 (not significant).	The small sample and short duration of this lifestyle education intervention had no effect and the results are not generalisable because of the small sample.
Polonsky et al., 2019, USA [69].	RCT, conducted in communities and schools.	1362 predominantly Black 4th through 6th grade students; Mean age = 10.8 years; 51% females.	A 2-year programme: Free school breakfasts and 18 45-min sessions of nutrition education, plus items with a healthy breakfast logo.	Control schools served breakfasts free of charge in the cafeterias before school and existing SNAP-Ed nutrition education continued.	There was no significant difference in the combined incidence of overweight and obesity between intervention schools (11.7%) and control schools (9.1%) after 2.5 years (odds ratio (OR) = 1.42; 95% CI = 0.82–2.44; *p* = 0.21).	Healthy school breakfasts and education alone without PA or home environment changes were not shown to be effective in preventing overweight or obese children. Moreover, the incidence of overweight and obese children was slightly higher in the intervention group.
Pena et al., 2022, USA [70].	RCT, conducted in community YCMA centres.	117 Hispanic youths; Aged 12 to 16, with prediabetes (fasting glucose 100 to 125 mg/dL or HbA1c level of 5.7% to 6.4%) and BMI >95th percentile for age and sex, according to CDC growth chart; 40% females.	A 52-week (12-month) programme: (i) One day/week of nutrition and health education with behaviour change skills training; (ii) Three days/week of physical activity.	Comparator group met with a paediatric endocrinologist and a bilingual, bicultural registered dietitian to discuss laboratory results and develop SMART goals for making healthy lifestyle changes.	The intervention led to significant decreases in mean 2-h glucose level (baseline: 144 mg/dL; 6 months: 132 mg/dL; *p* = 0.002) and increases in mean insulin sensitivity (baseline: 1.9 (0.2); 6 months: 2.6 (0.3); *p* = 0.001).	The 1-year education and structured PA intervention was effective in decreasing NCD metabolic risk in a high-risk group. However, there was no information on its effect on overweight/obesity.
Novotny et al., 2015, USA [71].	RCT, conducted in a clinical setting.	85 predominantly Asian children; Aged 5 to 8 years, with BMI between the 50th and 99th percentile for age and sex, according to CDC growth chart; 62% females.	A 39-week (9-month) programme: (i) Handout on recommended eating patterns and Dietary Approaches to Stop Hypertension (DASH) of Aloha cookbook; (ii) Farmers’ market locations; (iii) A PA location/map inthe study informational packet.	Received a welcome letter and attention control mailings on unrelated health topics, such as the importance of hand washing, sun protection and dental hygiene, at 2, 5 and 8 months.	There was no significant effect of the DASH intervention on changes in BMI z-score, SBP, waist circumference, total body fat by skinfolds, PA level or total Healthy Eating Index score (*p* > 0.05). DBP percentile was 12.2 points lower in the treatment group than the control group (*p* = 0.01).	The only effect was on DBP. However, as participants were in a clinical setting, there could have been ongoing clinical care.
Norman et al., 2016, USA [72].	RCT, conducted in a clinical setting.	106 predominantly Hispanic (82%) children; Aged 11–13 years, with BMI >95th percentile for age and sex, according to CDC growth chart; 51% females.	A 17-week (4-month) ‘steps’ programme, beginning with the most intensive contact followed by reduced contact if treatment goals were met. Based on the Chronic Care Model and social cognitive theory: (i) Counselling by a physician on healthy dietary and PA changes; (ii) Health educator visits to discuss weight management, barriers to healthy eating and PA; (iii) Follow-up phone calls.	Participants received an initial counselling visit by the physician, one visit with a health educator, materials on how to improve weight-related behaviours and monthly follow-up mailings on weight-related issues.	BMI (kg/m^2^) change differences between the intervention group and comparator: boys = 1.3, *p* = 0.003; girls = 0.7, *p* = 0.15. zBMI change differences between the intervention group and comparator: boys = 0.1, *p* = 0.008; girls = −0.2, *p* = 0.42. BF (kg): no difference (boys: *p* = 0.26; girls: *p* = 0.11. Fasting lipid profile and BP: no difference.	The intervention was shown to be effective in reducing BMI among boys but not girls. However, the intervention was tested in the age group of 11 to 13 years, a period of growth spurts in girls.
Messito et al., 2020, USA [46].	RCT, conducted in a clinical setting.	643 Hispanic pregnant mothers with a single uncomplicated pregnancy and postpartum infants; 54% of the infants were female.	33-month programme based on social cognitive theory to promote healthy behaviours: (i) Prenatal nutrition counselling; (ii) Postpartum lactation support; (iii) Nutrition and parenting support groups coordinated with paediatric visits.	Standard prenatal, postpartum and paediatric primary care.	Intervention infants had significantly lower mean weight for age z-scores at 18 months (0.49 vs. 0.73, *p* = 0.04) and 2 years (0.56 vs. 0.81, *p* = 0.03) but not at 3 years (0.63 vs. 0.59, (*p* = 0.76). Obesity prevalence was not significantly different between groups at any age point (33.5% vs. 39.4%, *p* = 0.11).	The intervention targeting mothers was only effective up to 18 months and was not sustained at 3 years.
Johnston et al., 2007, USA [73].	RCT, conducted in a school serving an urban student population.	60 Mexican American children; Aged between 10 and 14 years, with BMI ≥85th percentile for age and sex, according to CDC growth chart; 45% females.	A 6-month programme: (i) A 12-week instructor/trainer-led PA intervention 4 days per week, lasting 35 to 40 min at the school location; (ii) Nutrition instruction (1 day/week); (iii) Monthly meetings for parents to teach them how to adapt family meals and activities to facilitate healthy changes.	6-month parent-guided manual intended to promote child weight loss and the long-term maintenance of changes.	zBMI in the intervention group significantly reduced compared to the comparator group (F = 11.72, *p* < 0.001), with significant differences in zBMI at both 3 and 6 months (F = 16.50, *p* < 0.001 and F = 22.01, *p* < 0.001, respectively). Children in the intervention group significantly reduced their total cholesterol (F = 5.27, *p* = 0.027) and LDL cholesterol (F = 7.43, *p* = 0.01) compared to the children in the comparison group at 6 months.	In an urban setting, structured PA and nutrition was more effective than parental education alone over the short duration of 12 weeks. However, it was uncertain whether this improvement could be sustained long term.
Johnston et al., 2013, USA [74].	RCT, conducted in a school serving an urban student population.	71 Mexican American adolescents; Aged 10 to 14 years; 55% females.	12-week programme: 12 weeks of daily instructor/trainer-led healthy eating and PA behaviour change intervention sessions, followed by 12 weeks of biweekly follow-up sessions. Based on behaviour theory.	Given a parent-guided manual for the prevention and treatment of childhood obesity. The manual provided a 12-week weight management plan and instructions for the long-term maintenance of changes.	Repeated measures analyses revealed that adolescents in the intervention group significantly reduced their BMI z-scores compared to the adolescents in the control (F = 8.34, *p* < 0.001). Similar results for BMI (overall: F = 6.0, *p* < 0.01; 1 year: F = 6.6, *p* < 0.05; 2 years: F = 7.0, *p* < 0.05) and BMI percentile (overall: F = 5.8, *p* < 0.01; 1 year: F = 5.6, *p* < 0.05; 2 years: F = 6.6, *p* < 0.05). TC: F = 5.27, *p* = 0.027; LDL: F = 7.43, *p* = 0.01; HDL: F = 0.5, *p* > 0.05; TG: F = 0.5, *p* > 0.05.	Structured PA, nutrition education and long-term follow-ups were more effective than parental education in reducing both overweight/obesity and metabolic NCD risks. This effect was sustained for over 2 years.
Hull et al., 2018, USA [75].	RCT, conducted in a home setting in a metropolitan area.	318 Hispanic children; Aged 5 to 7 years, with at least one adult parent of Hispanic origin (self-identified) and BMI ≥25th <-35/kg/m^2^ percentile; 52% females.	52-week (12-month) programme aimed to increase PA, decrease sedentary behaviour and improve healthy eating behaviours. Used parental modelling and experiential learning for children. Based on social cognitive theory, behavioural choice theory and food preference theory.	Focused on oral health.	Intervention short-term effects: zBMI: 0.068, *p* = 0.11; BMI: 0.084, *p* = 0.42; WC to height ratio: −0.004, *p* = 0.15; WC to hip ratio: 0.005, *p* = 0.24. Intervention long-term effects: zBMI: 0.023, *p* = 0.25; BMI (Kg/m^2^): 0.067, *p* = 0.27; WC to height ratio: 0.006, *p* = 0.02; WC to hip ratio: −0.004, *p* = 0.15.	The purely education and behaviour change intervention showed no effect.
Hughes et al., 2021, USA [76].	RCT, conducted in community childcare centres.	25 predominantly Hispanic children; Aged 3 to 5 years; 50% females.	A 7-week programme: Weekly teaching sessions on nutrition and PA.	The control arm received no sessions.	BMI z-scores showed no significant changes (F = 0.18, *p* = 0.91).	Short duration nutrition education and infrequent PA showed no effect.
Hollar et al., 2010, USA [77].	Quasi-experimental with pre- and post-test analyses, conducted in elementary schools.	1197 predominantly Hispanic children; mean age = 7.8 years.	A 2-year programme: (i) Dietary intervention: Modifications to school breakfasts, lunches and extended-day snacks in the intervention schools; (ii) PA: Opportunities for PA during the school day.	Usual practice.	Significantly more children in the intervention schools stayed within the normal BMI percentile range for both years of the study than those in the control school (*p* = 0.02).	Long duration actual dietary changes and PA reduced BMI.
Heerman at al., 2019, USA [78].	RCT, conducted in physicians’ offices and community settings.	117 majority Hispanic child–parent pairs; Children aged 3 to 5 years and Spanish speaking, with BMI >50th percentile for age and sex, according to CDC growth chart; 54% females.	A 15-week programme: (i) Weekly 90-min education and PA sessions, followed by twice-monthly health coaching calls for 3 months.	The control group received a twice-monthly school readiness curriculum for 3 months.	After adjusting for covariates, the intervention effect on linear child BMI growth was −0.41 (kg/m^2^) per year (95% CI = −0.82 to 0.01, *p* = 0.05).	Surprisingly, health coaching alone showed effects in young children; however, the study was underpowered and not generalisable.
Hasson et al., 2012, USA [79].	RCT, conducted in a clinical setting.	100 African American and Latino children; Aged 14 to 18 years, with BMI > 95th percentile for age and sex, according to CDC growth chart; 61% females.	16-week programme: Intervention 1: Nutrition education once per week and four motivational interviews during the 16 weeks; Intervention 2: Nutrition + strength training: Participants also received strength training twice per week (~60 min/session) for 16 weeks supervised in a Human Laboratory.	Control group. pre- and post-intervention data were compared	Interventions were ineffective in reducing BMI, BMI z-score and BMI percentile, with no between group difference. Nutrition group reported better improvements in insulin sensitivity compared than the combined nutrition and strength training or Control groups (+16.5% vs. −32.3% vs. −6.9%, respectively; *p* < 0.01) and disposition index (+15.5% vs. −14.2% vs. −13.7%, respectively; *p* < 0.01). Hepatic fat fraction decreased by 27.3% in the Nutrition combined with Strength training group compared to 4.3% in the Control group and 0% in the Nutrition group, *p* < 0.01. Ethnicity by intervention interaction effects showed better response in Hispanic group to Nutrition intervention compared with African American who showed worsened fat mass and glucose control post intervention.	Neither type of the 16-week lifestyle intervention was effective in changing primary obesity BMI outcomes. However, interventions improved secondary metabolic outcomes (e.g. insulin sensitivity, hepatic fat, inflammation). Hispanic ethnicity responded well to nutrition education, which contrasted the counterproductive response found in African Americans. The latter may benefit from a more direct approach involving multiple exercise and nutritional components. Culturally contextualised and ethnically tailored lifestyle intervention approaches are needed.
Haines et al., 2016, USA [80].	RCT, conducted in community health centres and community agencies.	112 predominantly Hispanic parents/child dyads; Children aged 2–5 years; 50% females.	A 39-week programme: A total of 9 sessions on parenting skills, children’s education and homework assignments (based on social contextual framework theory).	Mailed publicly available educational materials on promoting healthy behaviours among preschoolers each week for 9 weeks.	BMI (kg/m^2^) decreased by a mean of 0.13 among children in the intervention arm and increased by 0.21 among children in the control arm, with an unadjusted difference of 20.34 (95% CI = 21.21, 0.53). After adjusting for child sex and age, the difference was minimal (20.36; 95% CI = 21.23, 0.51; *p* = 0.41).	The predominantly nutrition education programme was not effective on adiposity.
Gatto et al., 2017, USA [81].	RCT, conducted in elementary schools.	319 Hispanic children in 3rd, 4th and 5th grade in schools that offered after-school programmes.	12-week programme (LA Sprout). Weekly: (i) 45-min interactive cooking/nutrition lessons; (ii) 45-min gardening lessons; (iii) Parallel bimonthly classes were offered to parents. The intervention was based on self-efficiency theory.	Did not receive any nutrition, cooking or gardening information from investigators.	Intervention group had significantly greater reductions in BMI z-score than the control group (−0.1 (9.9%) vs. −0.04 (3.8%), respectively; *p* = 0.01). Intervention group had a 1.2 cm (1.7%) reduction in WC, while the control group had a 0.1 cm (0.1%) increase after the intervention (*p* < 0.001). Fewer children had metabolic syndrome (n = 1) after the intervention than before (n = 7), while the number of children in the control group with metabolic syndrome remained essentially the same between pre- (n = 3) and post-intervention (n = 4).	The predominantly school-based nutrition programme reduced both BMI and metabolic risks in the short term; however, it was not clear whether this could be sustained.
Fiechtner et al., 2021, USA [82].	RCT, conducted in clinical and community settings.	4044 Hispanic, low-income children; Aged 6 to 12 years, with BMI >85th percentile for age and sex, according to CDC growth chart; 48% females.	Two intervention groups: Intervention I: Healthy Weight Clinic: 30 h of multidisciplinary team nutrition and PA education for parents/guardians and children, alternating between group and individual sessions; Intervention II: YMCA Modified Healthy Weight and Your Child: A total of 25 education sessions were offered to parent/guardians and children over 1 year of age. Each session was 2 h long. Both groups were exposed to primary care provider weight management training and text messages to parents/guardians for self-guided behaviour change support.	Eight demographically matched comparison community health centres were chosen as control sites.	The mean difference in the % of children in the 95th percentile for BMI between Intervention II and intervention I was 0.75 (90% CI = 0.07 to 1.43), which did not support noninferiority. Compared to the control sites, children in Intervention I had a −0.23 (95% CI = −0.36 to −0.10) decrease in BMI (kg/m^2^) per year and there was a −1.03 (95% CI = −1.61 to −0.45) decrease in the % of children in 95th percentile for BMI. There was no significant effect on BMI in Intervention II.	There was no difference between offering the education (nutrition and PA) programme in a multidisciplinary clinical setting or a community YMCA setting in a large sample of low-income high-risk children. Both approaches reduced the percentage of children in the 95th percentile for BMI.
Eichner et al., 2016, USA [83].	Quasi-experimental with pre- and post-test analyses, conducted in a school setting.	353 predominantly America Indian children in 6th, 7th and 8th grade; Aged 12 to 15 years; 50% females.	A 5-year programme (Middle School Opportunity for Vigorous Exercise): (i) PA: walked or ran 1 mile each school day and then engaged in a team activity, such as basketball, soccer, football, dodgeball or volleyball.	Baseline data and second outcome data were collected from students who did not participate in the PA programme for comparison.	Mean BMI z-scores remained the same among girls participating in MOVE (from 0.7 to 0.7) and increased for nonparticipating girls (from 1.1 to 1.2). Mean BMI z-scores decreased among boys participating in MOVE (from 0.8 to 0.7) and increased among nonparticipating boys (from 1.1 to 1.2). Overall, MOVE participants had significantly lower BMI z-scores than nonparticipants (*p* = 0.01).	The 5-year PA programme was shown to prevent increases in BMI but as most of the children were not in the high-risk category, it did not reduce BMI z-scores.
Dos Santos et al., 2020, USA [84].	Quasi-experimental with pre- and post-test analyses, conducted in a school setting.	46 predominantly Hispanic parent–child dyads; Children aged from 10 to 16 years old, with a BMI ≥85th percentile for age and sex, according to CDC growth chart; 45% females.	An 8-week programme: (i) Joint education for parents and children on nutrition, PA and lifestyle issues; (ii) PA classes: Adolescents engaged in moderate to vigorous PA (e.g., lap runs).	None.	Mean BMI (kg/m^2^): Pre-intervention = 29.95 (SD = 5.82); post-intervention = 29.44 (SD = 5.78, *p* = 0.012). Participant waist–hip ratio from pre-intervention (mean = 1.00, SD = 0.06) to post intervention (mean = 0.99, SD = 0.06, *p* < 0.001).	Education and moderate PA had a small effect in reducing adiposity; however, the sample size was small and the intervention had a short duration. Long-term sustainability was uncertain.
De Heer et al., 2011, USA [48].	RCT, conducted in a school setting.	901 Hispanic students in 3rd, 4th and 5th grade; Mean age = 9.2 years; 45% females.	12-weeks after-school programme run twice weekly, based on social cognitive theory: (i) Education: 20- to 30-min health education component; (ii) PA: 45 to 60 min of PA.	Control and spill over groups received 4th grade health workbooks and incentives at pre-test and follow-up measurements, but they did not attend the after-school sessions.	BMI percentile reduction: Intervention group = 2.8% (*p* = 0.015); Spill over group = 2.0% (*p* = 0.085); Control group = 1.4% (*p* = 0.249).	This education and PA programme was shown to reduce BMI; however, a comparative analysis was not conducted. Therefore, it was unclear to what extent it was effective.
Davis et al., 2016, USA [85].	RCT, conducted in community and school settings.	1898 predominantly American Indian and Hispanic children; Aged 3 years, enrolled in Head Start (HS) centres; 47% females.	5-year programme based on a socioecological approach. The 6-component programme comprised nutrition and PA education and increasing the availability of healthier food options.	Participated in measurements but not the intervention.	No effect of the intervention on zBMI was observed: difference in slopes = −0.006 (95% CI = −0.031 to 0.020, *p* = 0.69).	This large and long-term predominantly prevention education intervention did not show any effect on BMI, although there were some reductions in zBMI.
Davis et al., 2012, USA [86].	RCT, conducted in schools, community centres and health clinics.	53 African American and Latino children in 9th through 12th grade; Mean age = 15.3 years, with BMI ≥85th percentile for age and sex, according to CDC growth chart; 55% females.	12-month maintenance programme (newsletter group) following a 4-month nutrition and strength training intervention: Received a monthly newsletter in the mail that matched their 4-month intervention group assignment.	Maintenance group class: Met monthly (classes lasted 90 min) and received a monthly class that was similar to the 4-month intervention classes.	Fasting insulin and acute insulin response decreased by 26% and 16% (*p* < 0.001 and *p* = 0.046), respectively, while HDL and insulin sensitivity improved by 5% and 14% (*p* = 0.042 and *p* = 0.039), respectively.	12-month programme of newsletters followed by nutrition and resistance training improved insulin and lipid metabolic profiles, although its effect on overweight/obesity was not assessed.
Davis et al., 2021, USA [87].	RCT, conducted in schools.	3135 predominantly Hispanic 3rd–5th grade students; Mean age = 9.2 years; 53% females.	9-month programme (Sprout): (i) Garden Leadership Committee formation; (ii) A 0.25-acre outdoor teaching garden; (iii) 18 student gardening, nutrition and cooking lessons; (iv) 9 monthly parent lessons. Based on a social ecological transactional model.	The control schools received a delayed intervention (identical intervention) in the year after the post-testing for that wave.	BMI change, mean (kg/m^2^): I = 4.12, C = 3.71, *p* = 0.006; BMI z-score change, mean: I = −0.04, C = −0.02, *p* = 0.51; BMI percentile change: I = −0.82, C = −0.39, *p* = 0.53; WC change, mean (cm): I = 1.16, C = −1.53, *p* = 0.34; % BF change: I = −0.34, C = −0.49, *p* = 0.40; SBP change, mean (mmHg): I = −0.39, C = 0.20, *p* = 0.64; DBP change, mean (mmHg): I = −1.33, C = 0.32, *p* = 0.18.	The nutrition intervention did not show any effects on most overweight/obesity parameters or blood pressure, except there was a difference in mean BMI change.
Davis et al., 2009, USA [47].	RCT, conducted in a clinical setting.	54 overweight Hispanic children; Aged 14 to 18 years (mean = 15.5), with BMI ≥85th percentile for age and sex, according to CDC growth chart.	16-week nutrition + strength training programme: In addition to the nutrition education classes described under comparator, participants in the intervention group also received strength training twice per week (~60 min/session) for 16 weeks.	Nutrition only group received culturally tailored dietary intervention once per week (~90 min) for 16 weeks.	There were no significant intervention effects on insulin sensitivity, body composition or most glucose/insulin indices, with the exception of glucose incremental area under the curve (*p* = 0.05), which decreased in the nutrition and nutrition + strength training groups by 18 and 6.3%, respectively, compared to a 32% increase in the control group.	The short duration and small size nutrition education and PA programme had no effects on adiposity or metabolic risk.
Davis et al., 2011, USA [88].	RCT, conducted in a school setting.	38 Hispanic females in grades 9–12; Aged 14 to 18 years, with BMI ≥85th percentile for age and sex, according to CDC growth chart.	16-week intervention: Intervention group I (circuit training): aerobic + strength training two times/week for 60–90 min per session; Intervention group II (circuit training + motivational interviews on behaviour change).	Control group was offered abbreviated circuit training intervention after post-test data collection.	No changes in BMI. Children in all groups increased their overall mean BMI z-score over the course of the study. Circuit training participants showed decreased waist circumference (−3% vs. +3%, *p* = 0.001). % BF: Subcutaneous adipose tissue (10% vs. 8%, *p* = 0.04) and visceral adipose tissue (10% vs. +6%, *p* = 0.05). Fasting insulin (24% vs. +6%, *p* = 0.03) and insulin resistance (−21% vs. −4%, *p* = 0.05).	The 16-week PA (aerobic and strength training) programme was effective in reducing fat depots and improving insulin resistance in Latino youths who were overweight/obese. The additional motivational interviews showed no additional benefits. Neither intervention had an effect on overweight/obesity.
Crespo et al., 2012, USA [89].	RCT, conducted in schools and community settings.	808 Hispanic parent–child dyads; Children mean age = 5.9 years; 50% females.	4-year intervention programme: Intervention I (Family group): Home visits (newsletters, recipe cards and goal setting) and follow-up phone calls; Intervention II (Community group): Improvements to nutrition and PA environments in school playgrounds and community parks. Distribution of education materials; Intervention 3 (Family + Community group): Involved in both family and community interventions.	Participants in the control group were asked to maintain their regular lifestyles and complete the yearly measurements.	No changes in any weight measures were statistically significant. Children in all groups increased their overall mean BMI z-score over the course of the study.	Despite the long duration, the nutrition education and support programme in family, school and combined family and school settings was not effective in reducing BMI. However, the effect of the intervention on other metabolic parameters was not assessed.
Chen et al., 2011, USA [90].	RCT, conducted in a web-based setting.	54 Chinese American adolescents; Aged 12 to 15 (mean = 12.5) years old and normal weight or overweight, with BMI ≥85th percentile for age and sex, according to CDC growth chart; 70% females.	An 8-week web-based programme (based on the transtheoretical model of stages of change): (i) Nutrition, PA and coping techniques; (ii) Internet sessions to coach parents on imparting the skills.	Participants in the control group also logged on to the website using a preassigned username and password. Every week for 8 weeks, adolescents also received general health information.	No reductions in BMI (Kg/m^2^) were observed in either the intervention (t0 = 20.79, T3 = 20.76) or control (t0 = 20.25, t3 = 20.21) groups. Significantly more adolescents in the intervention group decreased their waist to hip ratio than in the control group (Effect size = 0.01, *p* = 0.02). DBP (Effect size = 1.12, *p* = 0.02).	This short duration (8 weeks) web-based education programme was not shown to be effective in reducing BMI. However, as most of the participants were of normal weight, the intervention could have played a preventive role.
Chen et al., 2019, USA [91].	RCT, conducted in a community setting.	40 Chinese American children; Aged 13 to 18 years of age, with BMI ≥85th percentile for age and sex, according to CDC growth chart.	12-week intervention (based on social cognitive theory): (i) Used a wearable sensor (Fitbit Flex) for 6 months; (ii) Reviewed 8 online educational modules for 3 months and after completing the modules, they received tailored, biweekly text messages for 3 months.	After the completion of the baseline assessments, control group participants were given an Omron HJ-105 pedometer and a blank food and activity diary. The adolescents were asked to record and track their physical activity, sedentary activity and food intake in the diary for 3 months.	BMI (kg/m^2^) difference: −4.89 (*p* < 0.001); BMI z-score difference: −4.72 (*p* < 0.001).	With overweight/obese Chinese American children, the online education programme was effective in reducing BMI.
Chen et al., 2010, USA [92].	RCT, conducted in a community setting.	67 Chinese American children; Aged 8 to 10 years and were normal weight or overweight, with BMI ≥85th percentile for age and sex, according to CDC growth chart; 44% females.	An 8-week programme (based on social cognitive theory): (i) Children participated in a 45-min session of education- and play-based activities once each week for 8 weeks; (ii) Parents participated in two sessions that lasted 2 h each during the 8 weeks. The parents took part in ‘Healthy Eating and Healthy Family: A Hands-on Workshop’. Follow-up was 8 months.	Waiting-list control group received intervention after the follow-up period.	Significant decreases in BMI (kg/m^2^) were observed in the intervention group (19.74 to 19.32, *p* < 0.05) but not in the control group (18.65 to 18.42, *p* > 0.05). No changes in waist to hip ratio were reported in the intervention group (0.88 to 0.88, *p* > 0.05). Significant reductions in DBP were seen in the intervention group (61.03 to 59.27, *p* < 0.05).	Surprisingly, small reductions in BMI and CVD risk among Chinese American were shown after a few sessions of child and parent education and play activities. However, the results may not be generalisable because of convenient sampling.
Caballero et al., 2003, USA [93].	RCT, conducted in a school serving American Indian children.	1704 American Indian children in 3rd to 5th grade; Mean age = 7.6 years.	The 3-year intervention had 4 components: (i) Change in dietary intake; (ii) Increase in PA; (iii) A classroom curriculum focused on healthy eating and lifestyle; (iv) A family involvement programme.	The control group participated in measurements but not the intervention.	Mean differences at follow-up: %BMI: −0.2, *p* = 0.30; %BF: 0.2, *p* = 0.66; Triceps skinfold thickness (mm): 0.1, *p* = 0.84; Scapula skinfold thickness (mm): −0.1, *p* = 0.85.	Despite the long duration of implementation, the predominantly education and low-intensity PA programme did have effects on BMI/adiposity among American Indian children. However, there were indications that their calorie intake improved.
Barkin et al., 2011, USA [94].	RCT; participants were identified from primary care clinics, radio advertising and local churches.	72 mostly Hispanic parent–child dyads; Children aged 8 to 11 years, with BMI ≥85% for age and sex, according to CDC growth chart; 54% females.	6-month programme (based on a transtheoretical model): (i) Counselling by a physician trained in the brief principles of motivational interviewing; (ii) 45-min group health education session; (iii) 5 monthly 1-hour sessions on the topic of increasing PA for both parents and children.	Families in the control group received standard of care counselling from physicians trained using American Academy of Pediatrics guidelines, addressing both nutrition and activity.	Participants that had a higher baseline BMI were more likely to decrease their absolute BMI (Kg/m^2^) (β= −0.22, *p*< 0.0001).	The counselling and education were effective for obese children but less so for normal weight or overweight children.
Barkin et al., 2012, USA [95].	RCT, conducted in a community setting.	75 majority Hispanic parent–child dyads; Children aged 2 to 6 years; 48% females.	A programme: (i) Weekly 90-min skills-building sessions for parents and preschool-aged children designed to improve family nutritional habits, increase weekly PA and reduce sedentary activity.	A brief school readiness programme was conducted as an alternative to the active intervention because there was no standard care condition for comparison.	The effect of the treatment on post-intervention absolute BMI (Kg/m^2^) was B = –0.59 (*p* = 0.001).	This skills-building intervention programme targeting both parents and children with obesity had a small but significant effect on BMI.
Arlinghaus et al., 2017, USA [96].	RCT, conducted in a school setting.	189 Hispanic adolescent students in grades 6 through 12, with BMI ≥85% for age and sex, according to CDC growth chart; 47% females.	6-month programme: Trained peer-led discussion on the selected topic with their group of middle-school students during PE classes, e.g., what they were going to eat for lunch that day or their favourite vegetables.	Usual PE classes.	Significant differences were found between groups across time (F = 4.58, *p* = 0.01). After the 6-month intervention, the intervention group had a larger decrease in zBMI (F = 6.94, *p* = 0.01) than the control.	Adding peer-led nutritional education to PE classes reduced adiposity in high-risk Hispanic children.
Arlinghaus et al., 2021, USA [97].	RCT, conducted in a school setting.	491 Hispanic American middle-school students enrolled in PE classes; 53% females.	A 12-month programme: (i) The PA component of an obesity intervention with established efficacy at reducing standardised BMI among this population.	Control was physical education (PE) class as traditionally taught in the district.	Intervention decreased zBMI significantly more than the control (F (1, 56) = 6.16, *p* < 0.05).	The addition of the PA component to PE classes reduced overweight/obesity after 12 months; however, it was uncertain whether this was sustainable.
Adab et al., 2018, UK [45].	RCT, conducted in primary schools.	1392 non-White multi-ethnic children; Aged 5 to 6 years in year 1 of primary school; 51% females.	A 12-month programme: (i) Encouraged healthy eating and PA; (ii) Additional 30 min of school time every day for PA opportunities; (iii) A 6-week interactive skills-based programme in conjunction with a football club; (iv) Signposting of families to local PA places; (v) School-led family workshops on healthy cooking skills.	Ongoing health-related activities for pupils in year 2 study. In addition, citizenship education resources, excluding topics related to healthy eating and physical activity, were provided.	At 15 months, mean difference in BMI z-score: −0.075 (95% CI = −0.183 to 0.033, *p* = 0.18). At 30 months, mean difference: −0.027 (95% CI = −0.137 to 0.083, *p* = 0.63). There were no statistically significant differences between groups.	No significant effects of intervention on adiposity were observed in either the short or longer term. Although there were improvements in BMI, the differences were smaller the longer the duration of intervention.

**Abbreviations**: BF, body fat; BMI, body mass index; BP, blood pressure; DASH, Dietary Approaches to Stop Hypertension; BP, diastolic blood pressure; CDC, Centres for Disease Control; CI, confidence interval; C, control; FM, fat mass; FFM, free fat mass; HDL, high-density lipoprotein; HOM-AIR, Homeostatic Model Assessment of Insulin Resistance; I, intervention; LDL, low-density lipoprotein; NCD, noncommunicable disease; OGTT, oral glucose tolerance test; PA, physical activity; PE, physical education; RCT, randomised control trial; SBP, systolic blood pressure; SD, standard deviation; SE, standard error; TC, total cholesterol; TV, television; USA, United States of America; WC, waist circumference; YMCA, Young Men’s Christian Association.

### 3.3. Risk of Bias within Studies

All 53 studies were assessed for quality using the Cochrane risk of bias tool (random sequence generation for randomisation, allocation concealment, the blinding of participants and personnel, the blinding of outcome assessment and selective reporting) (see Supplementary Table S3, which contains full details of all studies). In total, 14 RCTs were deemed to have been conducted in a relatively unbiased manner, based on the Cochrane tool, and 15 RCTs were considered moderately biased, mostly because of a lack of a description of the blinding and allocation concealment processes. 

### 3.4. Effectiveness of Interventions

There were 44 RCT/controlled studies and 9 quasi-experimental pre- and post-intervention studies (Table 1). 

#### 3.4.1. Quasi-Experimental Pre- and Post-Intervention Studies

The results from the quasi-experimental studies were mostly effective intervention (seven of the nine studies) (Table 2). Eight studies measured BMI or other adiposity measures as the main outcome. Five studies reported significant effectiveness in reducing or maintaining BMI/zBMI/WC/%BF. Three of the studies that were shown to be effective in improving weight/obesity outcomes were conducted over a duration of more than 6 months and two were conducted for less than 6 months. In four of the studies, the interventions targeted children only, while one study targeted children and their families. Four of the studies were implemented in schools, two in community settings, one in a healthcare setting and one in combined healthcare and community settings. Four studies included measures of cardiometabolic risk factors, such as fasting glucose, blood pressure, insulin resistance and hepatic fats. Three [50,55,98] of the four studies showed significant effects in improving cardiometabolic risks.

#### 3.4.2. Randomised Control Studies

There were 44 RCTs that used PA, nutrition and behavioural interventions for the prevention of obesity and NCDs. In total, 40 of these studies (Table 1) included obesity measures, such as BMI, BMI z-score, BMI percentile, percentage body fat and waist circumference, as primary outcome measures, whereas 4 studies did not include obesity outcome measures. Of the 40 studies that examined obesity outcomes, 14 (35%) studies reported significant improvements in the outcomes, 8 of which were implemented among overweight/obese children (BMI ≥85th percentile for age and sex). Nine of the effective interventions targeted children only, while three targeted children and parents or families and two targeted parents only. In 8 of the 14 effective studies, the participants were more than 50% female and the settings were predominantly multiple settings and schools (6 of 14 studies).

There were 15 RCTs that examined the effects of interventions on cardiometabolic outcomes. Of the 15 effective RCTs, 11 (73%) showed effectiveness in improving cardiometabolic risk outcomes. In nine studies, participants were either overweight or obese and four studies had more than 50% female participants. Nine of the effective RCTs used combined PA, nutrition and behavioural interventions, six of which were implemented for more than 6 months. 

**Figure 2:**
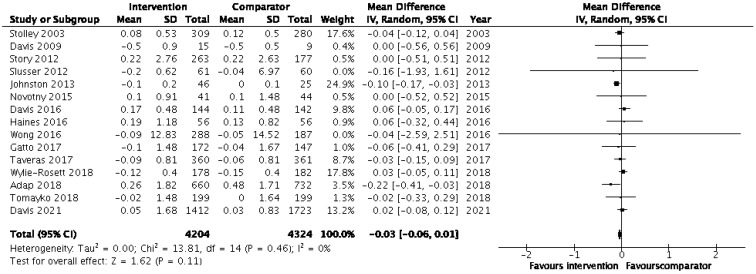
A forest plot of the change in mean BMI z-score following lifestyle interventions in children from minority ethnic groups [45,47,52,53,58,59,60,61,63,71,74,80,81,85,87].

**Figure 3:**
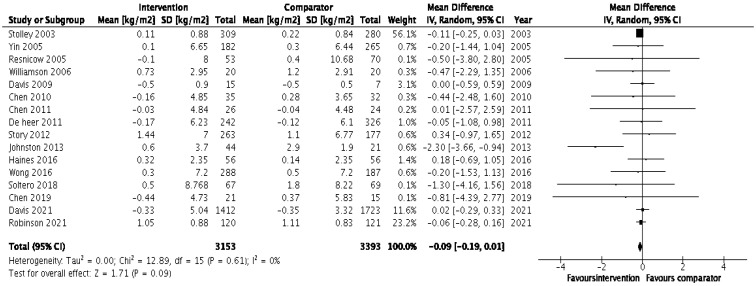
A forest plot of the change in mean BMI following lifestyle interventions in children from minority ethnic groups [47,48,51,53,56,60,61,62,65,67,75,81,88,91,92,93].

Overall, 15 RCTs were deemed suitable for the meta-analysis of the BMI z-score outcome and 16 for the BMI outcome (Figure 2 and Figure 3). The pooled effect of the 15 studies did not show any significant differences between intervention and comparator groups in terms of change in mean BMI z-score (−0.03 (95% CI = −0.06, 0.01)) (Figure 2). Similarly, the pooled effect of the 16 studies in the meta-analysis for BMI did not show any significant differences in terms of change in mean BMI (kg/m^2^) (−0.09 (95%CI = −0.19, 0.01)) (Figure 3). Although not significant, the pooled results were slightly in favour of intervention. However, according to our sensitivity analysis, none of the results changed significantly depending on the duration of implementation (<6 months vs. ≥6 months), the type of intervention (PA vs. nutrition/combined intervention) or weight status (overweight/obese vs. normal weight). The studies included in the meta-analyses for BMI z-score and BMI outcomes did not show significant heterogeneity. 

## 4. Discussion

### 4.1. Findings

This systematic review and meta-analysis examined data from 53 studies, involving 26045 children from minority ethnic groups in Western HICs who followed lifestyle preventative interventions for obesity and related comorbidities. The main finding of our meta-analysis was that lifestyle interventions were not significantly effective when they focused on BMI outcomes, which are commonly used as primary prevention outcomes. However, of the 53 RCTs included, there were 19 studies that reported reductions in BMI, BMI z-score and percentage body fat following lifestyle interventions in minority ethnic children. Furthermore, our analysis showed that constant adherence to supervised physical activity, diet and lifestyle interventions that adopted a direct approach using a quasi-experimental pre- and post- design were most effective, especially when they combined primary obesity outcomes with secondary comorbidity measures, including metabolic syndrome, insulin sensitivity and blood pressure. Therefore, incorporating obesity-related comorbidity NCD outcomes alongside BMI within lifestyle interventions could be essential for preventing the obesity-related complications of T2D, hypertension and CVD in high-risk overweight and obese ethnic minority children.

The interventions that were effective in ameliorating obesity and related comorbidities were mostly RCTs with low attrition, conducted under controlled settings (home, school, under parental supervision) and combined both PA and nutritional components [63,73,81,83,84,99]. For example, there was 7% reduction in BMI in Hispanic children when a 12-week instructor-led PA, nutrition and parental guidance programme was followed [100]. On the contrary, when interventions were remote, less supervised or carried out as compensation for a lack of PA, less or sometimes reverse effects were found. For example, BMI was either unchanged or increased with an intervention that provided handouts on recommended eating patterns (Aloha cookbook), farmers’ market locations and PA locations/maps [101]. This suggests that when targeting ethnic minorities, it is essential to consider supervised activities, such as instructor-guided PA, guided nutritional education and environmental changes. There is already evidence suggesting that culturally tailored and directly monitored interventions work better in minority ethnic communities [28,102]. Therefore, culturally acceptable nutrition and PA interventions that are embedded within usual supervised activities are feasible and more likely to be effective in preventing overweight and obese children among minority ethnic groups.

There are various potential explanations for the apparently weak evidence for the effectiveness of lifestyle interventions among children from minority ethnic groups living in Western HICs (Table 1). Firstly, most of the interventions were adapted from existing interventions to address cultural and socioeconomic barriers encountered by minority ethnic communities. However, for most minority ethnic groups, traditions from original homelands have a strong influence on physical activity and dietary practices while living in Western HICs [103]. Therefore, culturally tailored approaches are required. Previous reviews that examined diet and physical activity behaviours among adults from minority ethnic groups in HICs have similarly not found any concrete evidence for the significant effectiveness of diet and PA for the prevention of overweight and obesity people among minority ethnic groups [103]. For example, a review of the effects of diet and physical activity interventions on weight, BMI and waist circumference among South Asian migrants, including 29 studies, observed no significant differences in adiposity parameters, except for a significant improvement in weight (mean difference: 1.8 kg; 95% CI: 2.5 to 1.2 kg) [28].

Therefore, the development of effective interventions may require qualitative and quantitative research on the knowledge, attitudes, behaviours, perceptions and differential effects of lifestyle interventions among different ethnicities. Evidence for the effectiveness of such approaches was demonstrated in a study that developed interventions in collaboration with the Pakistani community using a social cognitive theory framework and taking into consideration the values, behaviours and perceptions of the targeted community [104]. Another successful approach, used in Chinese adolescents, was a culturally specific, family-based, interactive programme delivered in clinics (supervised) and online. The intervention entailed the early involvement of key community stakeholders and adolescents in the design and implementation of the intervention [105]. Therefore, simply adapting interventions developed for the general population may not be effective among specific minority ethnic groups, unlike those developed in partnership or that are culturally appropriate and implemented with the involvement of the entire family.

Variations in approaches adopted to supervise lifestyle interventions across different populations provide another possible explanation for the observed lack of effectiveness in children from some minority ethnic groups (Figure 2 and Figure 3). For example, African American children did not adhere well to an unstructured moderate-intensity school-based PA intervention combined with healthy snack education [51], despite the design and setting being effective when applied with other ethnic groups, such as American Indians and Hispanic groups [83,84]. The majority of the studies did not report the co-design of interventions with ethnic minorities groups, which is recommended to reduce disparities [20,23,24], thus limited their influence on intervention approaches. Therefore, there is an opportunity to improve interventions targeting minority ethnic groups by involving them in the design. The intervention that showed positive preventative effects involved a family setting and a combination of practical PA and nutrition interventions with adequate follow-up arrangements. Intervention type, setting, duration, frequency and follow-up varied among the studies included in this review. In terms of the intensity of PA, adolescent groups seem to benefit and adhere to more intense forms of vigorous PA compared to lower intensity forms of PA (Table 2). 

Despite the weak evidence for the benefits of lifestyle interventions on adiposity and BMI outcomes shown in our meta-analysis (pooled BMI mean change = −0.09 (95% CI = −0.19, 0.01, *p* = 0.09) (Figure 2 and Figure 3), lifestyle interventions were shown to be effective in reducing cardiometabolic NCD risk factors, such insulin sensitivity, metabolic syndrome and blood pressure, in overweight and obese children (Table 2). Over 70% of the studies in this review that examined cardiometabolic risk factors as secondary outcomes showed effectiveness (Table 1). Of the 11 studies that examined both BMI and metabolic risk factors as outcomes, only 5 (45%) showed impacts on overweight/obesity, whereas 9 (81%) showed effectiveness on cardiometabolic risks. Most of the effective interventions had multiple components with PA as the prominent part, whereas those that were not effective were mostly counselling and telephone follow-up interventions [52,72,87]. These data suggest that multicomponent lifestyle interventions may be effective in reducing metabolic risk faster than adiposity in the short term. Similar conclusions about the impacts of multicomponent programmes on lipid profile were made by Ho et al., 2012 [106]. They conducted a meta-analysis on the effects of lifestyle interventions on cardiometabolic outcomes, including 33 studies conducted among overweight and obese children, and found significant improvements in low-density lipoprotein cholesterol (−0.30 mmol/L, 95% CI = −0.45 to −0.15), triglycerides (−0.15 mmol/L, 95% CI = −0.24 to −0.07), fasting insulin (−55.1 pmol/L, 95% CI = −71.2 to −39.1) and blood pressure up to 1 year from baseline. Similarly, a meta-regression by El-Medany et al., 2019, showed significant improvements in SBP, LDL, TG and HDL (*p* < 0.05) with lifestyle interventions among 4- to 19-year-olds, with minimal changes in BMI SDS [107]. The quasi-experimental intervention designs we presented (Table 2) also showed improved lipid profile, blood pressure and cardiometabolic risk when multicomponent PA and nutrition interventions were adopted in children aged 11–18 years [50,52,55,108]. It was also noted that the most effective of those studies in targeting multiple conditions [52,109] were those with direct PA approaches that were able to tolerate higher intensity levels. For example, in one study [62], intense forms of exercise were well tolerated among obese adolescents, who improved their insulin sensitivity from 0.8 to 2.2 (*p* < 0.01) following 1 year of instructor-led structured and unstructured PA activities. It is likely that children with obesity-related comorbidities can tolerate and adhere to more intense forms of PA, especially under supervision, which can be readily provided throughout their lived environment by parents, coaches and teachers. Therefore, supervised games, sports and group activities are recommended for this high-risk group, which is in line with recent recommendations for the use of lifestyle interventions for the prevention of multiple long-term conditions [110]. 

There was paucity of studies that specifically targeted children from minority ethnic groups in other Western HICs except the USA. In this review, only one study was conducted in another Western HIC (the UK) [45]. This indicated a lack of targeted responses to the obesity and NCD scourge among children from minority ethnic groups in most Western HICs [111], even though it is known that obesity and NCD in children and adolescents is most prevalent among minority ethnic groups in HICs [112]. Therefore, considering that ethnic minorities have an increased risk of developing childhood obesity [113,114], it is critical that researchers develop effective interventions that can minimise such disparities. 

### 4.2. Implications for Practice and Research

We recommend that more obesity intervention studies targeting minority ethnic populations are conducted. These interventions should (1) be developed and implemented in partnership with minority ethnic communities, (2) be underpinned by appropriate and sound behavioural change techniques and theories and (3) be implemented with the involvement of the entire family. We also recommend that obesity-related comorbidity NCD risks, such as diabetes, hypertension and CVD, should be set as outcomes in addition to BMI and other adiposity measures in lifestyle intervention studies in high-risk children with obesity. 

These recommendations would enable reviewers to assess how behavioural change techniques and theories moderate effectiveness, assess the equity impacts of interventions and examine explanations for heterogeneity between interventions. More research into the differential effects of lifestyle interventions for specific ethnic minority groups compared to others is also required. 

### 4.3. Limitations

This review captured and analysed an extensive number of intervention studies and included a systematic analysis of many RCTs, on which a meta-analysis was conducted. While the included studies addressed interventions among minority ethnicities within Western HICs, emerging socioeconomic disparities within LMICs also require further research. While we attempted to highlight some of the emerging disparities elsewhere [21,115], further research is still needed on whether and how lifestyle interventions can be effective in reducing obesity-related comorbidities and health inequalities. 

## 5. Conclusions

Lifestyle interventions are essential for the prevention and management of obesity and associated comorbidities among children. Currently designed interventions focusing on reducing adiposity are not significantly effective in children from minority ethnic groups in Western HICs. Effective interventions in high-risk ethnic minority groups require jointly targeting obesity and its comorbidities as outcomes, especially diabetes, hypertension and cardiovascular disease, as well as actively engaging minority ethnic populations in the design and implementation. Public health policy makers and obesity prevention stakeholders should integrate cultural and lifestyle factors and contextualise obesity prevention strategies among minority ethnic groups in Western HICs.

## Figures and Tables

**Table 2:** Interventions with a quasi-experimental pre- and post- design.

Study, Year and Author	Intervention Type, Duration, Intensity and Time Characteristics	Intervention Setting	Age and Characteristics of Participating Children	Intervention Benefits on Obesity and Measured Comorbidity Outcomes	Recommendation for Effectiveness on Comorbidities
Effective interventions					
Yli-Piipari et al., 2018, USA [50].	12 weeks of supervised PA (moderate/vigorous, 60 min, twice a week) and nutrition education for parents/guardians.	Paediatric primary care setting.	Overweight/obese Hispanic children; Mean age = 11 years.	Change in mean BMI (kg/m^2^): −2.2, *p* = 0.04.	Short-term supervised high-intensity PA targeting high-risk adolescents was effective in reducing diabetes risk.
				Change in mean BMI%: −2.53, *p* = 0.02.	
				Change in mean BMI z-score: −3.64, *p* = 0.002.	
				Change in mean WC (cm): −2.57, *p* = 0.02.	
				Change in mean fasting glucose: −6.43, *p* < 0.001.	
Williford et al., 1996, USA [55].	15 weeks of supervised PA only (5 days/week, 45-min sessions of PE classes + conditioning programme).	School.	Predominantly African American children; Age range 12 to 13 (7th grade).	Change in sum of 7 skinfold thickness (mm): −1.31, *p* = 0.09.	Short-term, more frequent, moderate-intensity PA was effective in improving serum lipid profile, regardless of BMI.
				Change in mean HDL (mmol/L): 0.28, *p* < 0.05.	
				Change in mean LDL (mmol/L): −0.32, *p* < 0.05.	
Van der Heijden et al. 2010, USA [98].	12 weeks of supervised PA (twice a week, 30-min aerobic exercise session at ≥70% of peak oxygen consumption (VO2peak)).	Primary care (equipped laboratory in a hospital).	Lean and obese Hispanic children; Median age = 15 years.	Change in intrahepatic fats: Obese: −3.3, *p* < 0.05; Lean: No change.	Well-controlled short-term high-intensity exercise intervention was effective in reducing diabetes risk, only in high-risk obese children.
				Change in visceral fats: Obese: −5.1, *p* < 0.05; Lean: No change.	
				Change in fasting insulin: Obese: −3.6, *p* < 0.01; Lean: No change.	
				Change in HOM-AIR: Obese: −0.8, *p* < 0.01; Lean: No change.	
Rieder et al., 2013, USA [66].	6 months of supervised PA (60 min/week, moderate) and lifestyle education.	Community.	Mixed ethnic minority children; Mean age = 15 years.	Change in mean BMI (kg/m^2^): −0.7/month, *p* < 0.001.	Medium-term, moderate-intensity supervised PA was effective at the community level in adolescents with obesity.
				Change in mean BMI z-score: −0.003/month, *p* < 0.001.	
Hollar et al., 2010, USA [77].	2 years of unsupervised PA and dietary modifications.	School.	Predominantly Hispanic children; Mean age = 7.8 years.	Mean BMI: Maintained normal BMI, *p* = 0.02.	Longitudinal unsupervised PA with diet education was effective for the maintenance of healthy BMI in minority groups.
Eichner et al., 2016, USA [83].	5 years of unsupervised, unstructured PA (walk to school and team sports).	School.	Indian American children; Aged 12–15 years.	Change in BMI z-score: 0.7 to 0.7.	Very long duration, unsupervised, unstructured PA maintained BMI in American Indian adolescents.
Dos Santos et al., 2020, USA [84].	8 weeks of supervised PA (vigorous, e.g., lap runs).	School.	Overweight/obese mainly Hispanic children; Aged 10–16 years.	Change in BMI (kg/m^2^): −0.51, *p* = 0.012.	Short duration, supervised, high-intensity PA reduced obesity in high-risk Hispanic adolescents.
				Change in waist to hip ratio: −0.01, *p* < 0.001.	
**No significant effects of intervention**					
Yin et al., 2012, USA [49].	18 weeks of supervised, unstructured and structured PA (free play and 15 – 20 min PA), nutrition promotion and parental education.	Community centres and homes.	Hispanic children; Mean age = 4.1 years and range = 3 to 5 years.	Change in mean BMI z-score: −0.09, *p* = 0.09.	Short-term low-intensity PA and nutrition education was ineffective in very young Hispanic children.
Yin et al., 2005, USA [51].	24 weeks of intervention, including 129 days of after-school PA (moderate intensity, 80 min per week) and the provision of healthy snacks.	School (school and after-school sessions).	Predominantly African American children; Mean age = 8.7 years.	Change in mean BMI (kg/m^2^): 0.1, *p* > 0.05.	Short-term moderate-intensity PA was ineffective in reducing obesity or its comorbidities among African Americans.
				Change in %BF: −0.76, *p* = 0.027.	
				Change in FM (Kg): −0.29, *p* = 0.17.	
				Change in FFM (kg): 0.18, *p* = 0.12.	
				Change in mean WC (cm): −0.4, *p* = 0.32.	
				Change in mean SBP (mmHg): −1.8, *p* = 0.15.	
				Change in mean DBP (mmHg): −1.1, *p* = 0.41.	
				Change in mean TC (mg/dL): −0.2, *p* = 0.94.	
				Change in mean HDL (mg/dL): 0.7, *p* = 0.64.	

## Data Availability

The data are available from the authors upon request.

## Supplementary Materials

The following supporting information can be downloaded at https://www.mdpi.com/article/10.3390/nu15112524/s1, Table S1: MEDLINE search strategy with RCT filter; Table S2: Screening checklist; Table S3: Risk of bias of selected studies; Table S4: Data extraction form.
