# Supplementary material for: Effectiveness of Lifestyle Nutrition and Physical Activity Interventions for Childhood Obesity and Associated Comorbidities among Children from Minority Ethnic Groups: A Systematic Review and Meta-Analysis
Source: Nutrients. 2023 May 29;15(11):2524. doi: 10.3390/nu15112524 (PMC10255126; doi:10.3390/nu15112524)
Supplement: Supplementary file 1 [file nutrients-15-02524-s001.zip › nutrients-2411425-supplementary.pdf]

Supplementary Tables:

1. **MEDLINE search strategy with RCT filter**

#	**Query**
**Children (0 – 18)**	
S1	child*
S2	adolescen*
S3	pediatric
S4	paediatric
S5	student
S6	pupils
S7	youth
S8	boys
S9	girls
S10	teen*
S11	"school age*"
S12	juvenile
S13	"pre-teen*"
S14	preteen*
S15	(MH "Child+")
S16	(MH "Adolescent")
S17	(MH "Pediatrics+")
S18	(MH "Students")
S19	S1 OR S2 OR S3 OR S4 OR S5 OR S6 OR S7 OR S8 OR S9 OR S10 OR S11 OR S12 OR S13 OR S14 OR S15 OR S16 OR S17 OR S18
**Minority ethnic group**	
S20	BME
S21	BAME
S22	(MH "Ethnic and Racial Minorities")
S23	(MH "Blacks")
S24	(MH "Asians")

S25	(MH "Hispanic or Latino")
S26	black and minority ethnic
S27	black african
S28	indian
S29	pakistani
S30	bangladeshi
S31	chinese
S32	hispanic
S33	"mixed race"
S34	"mixed ethnicit*"
S35	S20 OR S21 OR S22 OR S23 OR S24 OR S25 OR S26 OR S27 OR S28 OR S29 OR S30 OR S31 OR S32 OR S33 OR S34
S36	S19 AND S35
**Physical activity**	
S37	(MH "Exercise+")
S38	(MH "Sports+")
S39	(MH "Bicycling")
S40	(MH "Walking+")
S41	(MH "Physical Education and Training")
S42	(MH "Dance Therapy")
S43	physical activity
S44	exercise
S45	sport
S46	cycling
S47	walking
S48	physical education
S49	aerobics
S50	fitness N5 (class or regime or program*)
S51	dance therapy
S52	movement therapy

S53	S37 OR S38 OR S39 OR S40 OR S41 OR S42 OR S43 OR S44 OR S45 OR S46 OR S47 OR S48 OR S49 OR S50 OR S51 OR S52
**Diet/Nutrition**	
S54	(MH "Diet Therapy+")
S55	(MH "Diet+")
S56	(MH "Fasting")
S57	(MH "Diet, Healthy")
S58	(MH "Food, Formulated")
S59	diet
S60	dieting
S61	fasting
S62	healthy eating
S63	eating fruits and vegetables
S64	formula diet
S65	S54 OR S55 OR S56 OR S57 OR S58 OR S59 OR S60 OR S61 OR S62 OR S63 OR S64
**Psychosocial intervention**	
S66	(MH "Behavior Therapy+")
S67	(MH "Social Support+")
S68	(MH "Psychotherapy, Group+")
S69	(MH "Family Therapy")
S70	(MH "Counseling+")
S71	(MH "Health Education+")
S72	(MH "Health Promotion+")
S73	(MH "Health Policy+")
S74	(behavior or behaviour*) N5 therapy
S75	social support
S76	(group or family) N5 (psychotherapy or therapy or intervention)
S77	counselling or counseling
S78	peer support

S79	Health Education
S80	health promotion
S81	media intervention
S82	community intervention
S83	school program*
S84	health policy N5 (food or nutrition)
S85	S66 OR S67 OR S68 OR S69 OR S70 OR S71 OR S72 OR S73 OR S74 OR S75 OR S76 OR S77 OR S78 OR S79 OR S80 OR S81 OR S82 OR S83 OR S84
S86	S53 OR S65 OR S85
**Obesity**	
S87	(MH "Obesity+")
S88	(MH "Body Constitution+")
S89	(MH "Weight Gain+")
S90	(MH "Body Fat Distribution")
S91	(MH "Adiposity")
S92	(MH "Body Mass Index")
S93	(MH "Body Size+")
S94	(MH "Waist Circumference+")
S95	(MH "Waist-Height Ratio")
S96	(MH "Body Surface Area")
S97	(MH "Skinfold Thickness")
S98	(MH "Waist-Hip Ratio")
S99	(MH "Overweight+")
S100	obesity
S101	body constitution
S102	body weight increase
S103	body fat distribution
S104	adiposity
S105	body mass index

S106	bmi
S107	body size
S108	abdominal circumference
S109	waist height ratio
S110	body surface
S111	skinfold thickness
S112	waist hip ratio
S113	overweight
S114	"over weight"
S115	neck circumference
S116	S87 OR S88 OR S89 OR S90 OR S91 OR S92 OR S93 OR S94 OR S95 OR S96 OR S97 OR S98 OR S99 OR S100 OR S101 OR S102 OR S103 OR S104 OR S105 OR S106 OR S107 OR S108 OR S109 OR S110 OR S111 OR S112 OR S113 OR S114 OR S115
**Obesity associated NCDs**	
S117	(MH "Dyslipidemias+")
S118	(MH "Asthma")
S119	(MH "Anxiety")
S120	(MH "Non-alcoholic Fatty Liver Disease")
S121	(MH "Depression")
S122	(MH "Self Concept+")
S123	(MH "Heart Diseases+")
S124	(MH "Cardiovascular Diseases+")
S125	(MH "Hypertension")
S126	(MH "Metabolic Syndrome")
S127	dyslipidemia
S128	respiratory problems
S129	asthma
S130	anxiety
S131	psychological problems
S132	non-alcoholic fatty liver disease

S133	nonalcoholic fatty liver disease
S134	nafld
S135	metabolic syndrome
S136	sleep disorders
S137	depression
S138	self esteem
S139	selfesteem
S140	self concept
S141	heart disease
S142	cardiovascular disease
S143	cvd
S144	high blood pressure
S145	hypertension
S146	(MH "Diabetes Mellitus, Type 2+")
S147	MODY or NIDDM or T2DM or T2D
S148	"non insulin* depend*" or "noninsulin* depend*" or "non insulindepend*" or noninsulindepend*
S149	("type-2" or "typ-2" or "type 2" or "typ 2" or "typ2" or "type2" or "type-ii" or "typ-ii" or "type ii" or "typ ii" or "typii" or "typeii") N6 diabet*
S150	((late or adult* or matur* or slow or stabl*) N3 (onset)) AND diabet*
S151	S117 OR S118 OR S119 OR S120 OR S121 OR S122 OR S123 OR S124 OR S125 OR S126 OR S127 OR S128 OR S129 OR S130 OR S131 OR S132 OR S133 OR S134 OR S135 OR S136 OR S137 OR S138 OR S139 OR S140 OR S141 OR S142 OR S143 OR S144 OR S145 OR S146 OR S147 OR S148 OR S149 OR S150
S152	S116 OR S151
S153	S36 AND S86 AND S152
**RCT Filters**	
S154	PT "Randomized Controlled Trial"
S155	PT "Controlled Clinical Trial"
S156	AB randomized
S157	AB placebo

S158	SU "Clinical Trials as Topic"
S159	AB randomly
S160	TI trial
S161	S154 or S155 or S156 or S157 or S158 or S159 or S160
S162	MH "Animals+" NOT SU "Humans"
S163	S161 NOT S162
S164	S153 AND S163

2. Screening checklist

**Criteria**
Study design
RCT
Quasi RCT
Feasibility
Population
0 - 18
Minority ethnic group
Intervention
PA
Diet
Reduction in sedentary activities
Psychosocial intervention
Comparator
No intervention/placebo
Other intervention
Other population groups
Outcomes
Obesity reduction measures
Other weight measures
PA level change
Change in dietary lifestyle
Change in sedentary lifestyle
Setting
HIC - western countries
Language
English
Other – translatable to English via google translate

3. Risk of bias of selected studies

Study	Randomisation	Allocation concealment	Blinding of participants and personnel	Blinding of outcome assessment	Incomplete outcome data	Selective reporting
Yli-Piipari et al., 2018, USA	**High risk** A quasi-experimental design	**High risk** A quasi-experimental design	**High risk** A quasi-experimental design	**High risk** A quasi-experimental design	**Unclear risk** No information	**Low risk** Reported as planned
Yin et al., 2012, USA	**High risk** A quasi-experimental design	**High risk** A quasi-experimental design	**High risk** A quasi-experimental design	**High risk** A quasi-experimental design	**Low risk** 88% completed. Second data collection	**Low risk** Reported as planned
Yin et al., 2005, USA.	**High risk** A quasi-experimental design	**High risk** A quasi-experimental design	**High risk** A quasi-experimental design	**High risk** A quasi-experimental design	**Low risk** More than 90% completed. Second data collection	**Low risk** Reported as planned
Wylie-Rosett et al., 2018, USA.	**Low risk** "Sequentially numbered, opaque, sealed envelopes to randomize families based on a computer generated 1:1 allocation schedule created by the data unit"	**Unclear risk** No information	**Unclear risk** No information	**Unclear risk** No information	**Low risk** 100% completed. Second data collection	**Low risk** Reported as planned
Wong et al., 2016, USA.	**Unclear risk** No information	**Unclear risk** No information	**Unclear risk** No information	**Unclear risk** No information	**High risk** 54% of participants to final data collection	**Low risk** Reported as planned
Wilson et al., 2022, USA.	**Low risk** "Using a computer-generated randomized algorithm"	**Unclear risk** No information	**Unclear risk** No information	**Unclear risk** No information	**Unclear risk** No information	**Unclear risk** No information
Williford et al., 1996, USA.	**High risk** A quasi-experimental design	**High risk** A quasi-experimental design	**High risk** A quasi-experimental design	**High risk** A quasi-experimental design	**Unclear risk** No information	**Low risk** Reported as planned
Williamson et al., 2006, USA.	**Low risk** "Randomly as- signed to the treatment arms using a stratified randomization strategy based on BMI percentile and age"	**Unclear risk** No information	**Unclear risk** No information	**Unclear risk** No information	**High risk** About 30% attrition	**Low risk** Reported as planned
Van der Heijden et al. 2010, USA.	**High risk** A quasi-experimental design	**High risk** A quasi-experimental design	**High risk** A quasi-experimental design	**High risk** A quasi-experimental design	**Unclear risk** No information	**Low risk** Reported as planned
Tomayko et al., 2018, USA.	**Low risk** "Randomization was conducted using a permuted block strategy prepared by the study biostatistician"	**Unclear risk** No information	**Unclear risk** No information	**Unclear risk** No information	**High risk** dropout was 16.4%	**Low risk** Reported as planned
Taveras et al., 2017, USA.	**Low risk** "We randomized participants using 6 separate randomization lists"	**Unclear risk** No information	**Unclear risk** No information	**Unclear risk** No information	**Low risk** All randomised participants had their data analyses	**Low risk** Reported as planned

Story M et al., 2012, USA.	**Low risk** "Schools were randomized to intervention and control conditions following baseline data collection"	**Unclear risk** No information	**Unclear risk** No information	**Unclear risk** No information	**Unclear risk** No information	**Low risk** Reported as planned
Stolley et al., 2003, USA.	**Unclear risk** No information	**Unclear risk** No information	**Unclear risk** No information	**Unclear risk** No information	**Low risk** 95% completed Second data collection	**Low risk** Reported as planned
Soltero et al., 2018, USA.	**Low risk** "Randomized by a research team member using the automated random sample function in SPSS"	**Unclear risk** No information	**Unclear risk** No information	**Unclear risk** No information	**Low risk** "2-month follow-up period for all youth was 82.5%"	**Low risk** Reported as planned
Slusser et al., 2012, USA.	**Unclear risk** No information	**Unclear risk** No information	**Unclear risk** No information	**Unclear risk** No information	**Low risk** 84% of follow up data available	**Low risk** Reported as planned
Shaibi et al., 2006, USA. s.	**Unclear risk** No information	**Unclear risk** No information	**Unclear risk** No information	**Unclear risk** No information	**Unclear risk** No information	**Low risk** Reported as planned
Robinson et al., 2021, USA.	**Low risk** "Households were randomized by computer by a study statistician to the MMM or Health Education conditions"	**Unclear risk** No information	**Unclear risk** No information	**Unclear risk** No information	**Low risk** "Retention was high, 238 (98.8%) over 1 year, 233 (96.7%) over 2 years, and 227 (94.2%) over 3 years, with 225 (93.4%)"	**Low risk** Reported as planned
Rieder et al., 2013, USA. .	**High risk** A quasi-experimental design	**High risk** A quasi-experimental design	**High risk** A quasi-experimental design	**High risk** A quasi-experimenta l design	**High risk** "Ninety-one participants (26%) completed the 9-month program"	**Low risk** Reported as planned
Resnicow et al., 2005, USA.	**Unclear risk** No information	**Unclear risk** No information	**Unclear risk** No information	**Unclear risk** No information	**Low risk** "A total of 147 girls completed the baseline assessment, from whom 6-month follow-up data were available for 123 (84%)"	**Low risk** Reported as planned
Prado et al., 2020, USA.	**Low risk** "Using urn randomization[29] and concealment of allocation procedures"	**Low risk** "Using urn randomizatio n[29] and concealment of allocation procedures"	**Low risk** "Single-blinded"	**High risk** single-blinded	**Low risk** "5%" attrition	**Low risk** Reported as planned
Polonsky et al., 2019, USA.	**Low risk** "Schools were randomized within pairs by using a random number generator"	**Unclear risk** No information	**Unclear risk** No information	**Unclear risk** No information	**High risk** "By the end of the study, 793 students (58.2%) remained in the study"	**Low risk** Reported as planned

Pena et al., 2022, USA.	**Low risk** “Youths were randomized in blocks of masked size using a 2:1 ratio (INT:UCC) with the automated random sample function in SPSS statistical software version”	**High risk** “It was not possible to mask each participant’s treatment group”	**High risk** “It was not possible to mask each participant’s treatment group”	**Low risk** “But outcome assessors were masked”	**High risk** “Median attendance was 63% (IQR, 30%-75%) for nutrition classes and 75% (IQR, 25%-88%) for physical activity classes, while 28 youths (74%) attended both usual care visits”	**Low risk** Reported as planned
Novotny et al., 2015, USA.	**Low risk** “Random number assignment from random.org”	**Unclear risk** No information	**Unclear risk** No information	**Unclear risk** No information	**Unclear risk** No information	**Low risk** Reported as planned
Norman et al., 2016, USA.	**Low risk** “Independent of randomization assignment”	**Unclear risk** No information	**Unclear risk** No information	**Unclear risk** No information	**High risk** Because there was attrition of 27% (n = 23) of children at 9 months and 31% (n = 26) at 15 months	**Low risk** Reported as planned
Messito et al., 2020, USA.	**Low risk** “Using computer-generated random numbers”	**Unclear risk** No information	**Unclear risk** No information	**Unclear risk** No information	**High risk** “Clinical measurements were available for 358 (67.7%) children at 2 years and 285 (53.9%) children at 3 years”	**Low risk** Reported as planned
Johnston et al., 2007, USA.	**Low risk** “Random allocation sequence using SPSS 13 statistical software”	**Unclear risk** No information	**Unclear risk** No information	**Unclear risk** No information	**Low risk** “57 of 60 completers analysed”	**Low risk** Data collected from all completers
Johnston et al., 2013, USA.	**Unclear risk** Randomisation method not described. “The original sample consisted of 71 overweight or obese students, 46 of whom were randomized to the instructor-led intervention (ILI), and 25 to a self-help (SH) condition.”	**Unclear risk** No information	**Unclear risk** No information	**Unclear risk** No information	**Low risk** 82% completed measurement	**High risk** “All students regardless of weight classification were eligible to participate in the study; however, only the overweight and obese participants were included in analyses”
Hull et al., 2018, USA.	**Low risk** “Generated the random allocation sequences using a computerized random number generator.”	**High risk** Not possible to mask participants or interventionists (CHPs) to group assignment	**High risk** Not possible to mask participants or interventionists (CHPs) to group assignment	**Low risk** interviewers are masked to group assignment	**High risk** “For short-term follow-up, 168 families (62%) with 206 children were included in the statistical analyses, along with 142 families (52.3%) with 169 children for long-term follow-up.	**Low risk** Of the 318 participating children (271 85% families), 254 (213 families) had any follow-up data,

Hughes et al., 2021, USA.	**Low risk** “Computer-based randomisation tool to assign participants to 1 of 2 arms using simple randomisation”	**Unclear risk** No information	**Unclear risk** No information	**Low risk** “Data collectors were blinded to participant group allocation”	**Unclear risk** No information	**Unclear risk** No information
Hollar et al., 2010, USA.	**High risk** “In a quasi-experimental design schools were nonrandomly assigned”	**High risk** A quasi-experimental design	**High risk** A quasi-experimental design	**High risk** A quasi-experimenta l design	**Low risk** Data complete	**Low risk** Reported as planned
Heerman at al., 2019, USA.	**Low risk** randomized to either the intervention or control group using a sequence with randomly permuted blocks of varying size.	**Unclear risk** Because this is a behavioural intervention, neither the interventionis t nor the participants could be blinded to study group assignment	**Unclear risk** Because this is a behavioural intervention, neither the interventioni st nor the participants could be blinded to study group assignment	**Low risk** All study staff who collect or interpret data are blinded to study group assignment	**Low risk** 100% allocated participants had data analysed	**Low risk** “Adjusted BMI difference estimates at each follow-up time point”
Hasson et al., 2012, USA.	**Low risk** “Randomisation was blocked by sex to achieve balance in randomization between sexes.”	**Unclear risk** No information	**Unclear risk** No information	**Unclear risk** No information	**Low risk** 79% of participants had data analysed	**Low risk** All participant who completed had data reported
Haines et al., 2016, USA.	**Low risk** “We used a stratified block randomisation scheme; stratum was site”	**High risk** “Neither the participants nor the research staff was blinded to the families’ intervention status.”	**High risk** “Neither the participants nor the research staff was blinded to the families’ intervention status.”	**High risk** “Neither the participants nor the research staff was blinded to the families’ intervention status.”	**Low risk** 86% had data collect	**Low risk** All relevant data reported
Gatto et al., 2017, USA.	**Unclear risk** No information	**Unclear risk** No information	**Unclear risk** No information	**Unclear risk** No information	**Low risk**	**Low risk**
Fiechtner et al., 2021, USA. .	**Low risk** We use a simple randomization by FQHC site	**Unclear risk** No information	**Unclear risk** No information	**Low risk** coordinators blinded to intervention assignment	**Low risk** Outcome assessed in 92% and 76% of each arm	**Low risk** Relevant data reported
Eicherner et al., 2016, USA.	**High risk** A quasi-experimental design	**High risk** A quasi-experimental design	**High risk** A quasi-experimental design	**High risk** A quasi-experimenta l design	**Unclear risk** No information	**Unclear risk** No information
Dos Santos et al., 2020, USA.	**High risk** A quasi-experimental design	**High risk** A quasi-experimental design	**High risk** A quasi-experimental design	**High risk** A quasi-experimenta l design	**Low risk** 84% completed post intervention data collection	**Low risk** All relevant data collected
De Heer et al., 2011, USA.	**Low risk** Randomization of the intervention occurred on a classroom level	**Unclear risk** No information	**Unclear risk** No information	**Unclear risk** No information	**Low risk** 90% completed data collection	**Low risk** Relevant outcome reported

Davis et al., 2016, USA.	**Low risk** “A random uniform variable was generated for each HS center”	**Unclear risk** No information	**Unclear risk** No information	**Unclear risk** No information	**Low risk** 95% of enrolled participants data analysed	**Low risk** Relevant outcome reported
Davis et al., 2012, USA.	**Low risk** “Randomization was conducted by a statistician independent from the study using computer-generated random numbers”	**Unclear risk** No information	**Low risk** “allocations were concealed from participants until after they consented to be in the maintenance programme”	**Unclear risk** No information	**Low risk** “61 participants allocated to the maintenance programme and the 53 who actually completed it.”	**Low risk** Relevant outcome reported
Davis et al., 2021, USA.	**Low risk** “Randomly assigned to either the intervention (n=8 schools) or control group (delayed intervention; n=8 school)’	**Unclear risk** No information	**Unclear risk** No information	**Unclear risk** No information	**Low risk** 13% lost to follow up	**Low risk** Relevant outcome reported
Davis et al., 2009, USA.	**Low risk** “Randomization was blocked by gender to achieve balance in randomization between genders”	**Unclear risk** No information	**Low risk** “Allocations were concealed from participants until after their in-patient visit”	**Unclear risk** No information	**Low risk** 79% of allocated participants completed data collection	**Low risk** Relevant outcome reported
Davis et al., 2011, USA.	**Low risk** “Randomization was blocked by intervention group to achieve balance in randomization”	**Low risk** “Allocations were concealed from participants until after pretesting”	**Unclear risk** No information	**Unclear risk** No information	**Low risk** 38 of 44 completed the programme	**Low risk** Relevant outcome reported
Crespo et al., 2012, USA.	**Low risk** “Randomization of schools to study conditions took place immediately after all participants completed baseline measures”	**Unclear risk** No information	**Unclear risk** No information	**Unclear risk** No information	**High risk** High drop out rate at final outcome measurement (52% -41%)	**Low risk** Relevant outcome reported
Chen et al., 2011, USA.	**Low risk** “Randomly assigned to the intervention group or the control group on the basis of a computer-generated random number assignment”	**Unclear risk** No information	**Unclear risk** No information	**Unclear risk** No information	**Low risk** 7% drop out rate	**Low risk** Relevant outcome reported
Chen et al., 2019, USA.	**Low risk** A randomization table that was stratified by gender using the IBM SPSS program was used	**Unclear risk** No information	**Unclear risk** No information	**Unclear risk** No information	**Low risk** 10% drop out rate	**Low risk** Relevant outcome reported

Chen et al., 2010, USA.	**Unclear risk** No information	**Unclear risk** No information	**Unclear risk** No information	**Unclear risk** No information	**Unclear risk** No information	**Low risk** Relevant outcome reported
Caballero et al., 2003, USA.	**Unclear risk** No information	**Unclear risk** No information	**Unclear risk** No information	**Unclear risk** No information	**Low risk** 17% lost to follow-up	**Low risk** Relevant outcome reported
Barkin et al., 2011, USA.	**Unclear risk** No information	**Unclear risk** No information	**Unclear risk** No information	**Unclear risk** No information	**High risk** 32% drop out rate	**Low risk** Relevant outcome reported
Barkin et al., 2012, USA.	**Low risk** "A computer-generated permuted block randomization scheme with a block size of 10 was used to ensure balanced treatment allocation once the total sample size was reached"	**High risk** "Neither research staff nor participants were blinded to other participants' condition allocation"	**High risk**	**High risk**	**Low risk** 7% lost to follow-up/data not analysed	**Low risk** Relevant outcome reported
Arlinghaus et al., 2017, USA.	**Unclear risk** No information	**Unclear risk** No information	**Unclear risk** No information	**Unclear risk** No information	**High risk** 25% drop out	**Low risk** Relevant outcome reported
Arlinghaus et al., 2021, USA.	**Unclear risk** No information	**Unclear risk** No information	**Unclear risk** No information	**Unclear risk** No information	**Low risk** 8% lost to follow-up/data not analysed	**Low risk** Relevant outcome reported
Adap et al., 2018, UK.	**Low risk** "Randomization is at the level of the cluster using a random number generator"	**Unclear risk** No information	**Unclear risk** No information	**Unclear risk** No information	**Low risk** 5% lost to follow-up/data not analysed	**Low risk** Relevant outcome reported

4. Data extraction form

**Criteria**
**1. Study details**
• Study title
• Authors
• Source
• Year
• Country
• Setting
• Site
• Study aim
• Study design
• Study question
• RCT design
○ How was blinding carried out?
○ Allocation concealment
○ Method of randomization
○ Power calculation
○ Dropout rate (%)

**2.**	**Participants characteristics**
	• Age range
	• Median age
	• Sex distribution
	• Sample size
	• Stratification
	• Inclusion
	• Exclusion
**3.**	**Intervention details**
	• Type of lifestyle intervention
	• Dose of Intervention if applicable
	• Frequency of administration
	• Duration of intervention
	• Method implementation of intervention
**4.**	**Comparator details**
	• Type of comparator
	• Dose, if applicable
	• Frequency of administration
	• Duration if applicable
	• Method implementation
**5.**	**Outcome**
	• Type primary outcome used
	• Outcome measures and units
	• Types of secondary outcomes use
	• Outcome measures for secondary outcome
**6.**	**Analysis**
	• Statistical methods used
**7.**	**Main results**
**8.**	**Conclusion**
**9.**	**Comments**
**10.**	**Limitations**
